# Abnormal α-synuclein binds to synaptotagmin 13, impairing extracellular vesicle release in synucleinopathies

**DOI:** 10.1186/s40035-025-00493-6

**Published:** 2025-06-23

**Authors:** Yasuo Miki, Shuji Shimoyama, Makoto T. Tanaka, Hanae Kushibiki, Asa Nakahara, Xiaopeng Wen, Masanori Hijioka, Tomoya Kon, Megha Murthy, Tomonori Furukawa, Conceição Bettencourt, Fumiaki Mori, Hiroki Mizukami, Shirushi Takahashi, Mari Tada, Yoshihisa Kitamura, Akiyoshi Kakita, Thomas T. Warner, Koichi Wakabayashi

**Affiliations:** 1 Department of Neuropathology, Biomedical Research Center, Hirosaki University Graduate School of Medicine, Hirosaki, 036-8562 Japan; 2 Department of Neurophysiology, Biomedical Research Center, Hirosaki University Graduate School of Medicine, Hirosaki, 036-8562 Japan; 3 Department of Pathology and Molecular Medicine, Biomedical Research Center, Hirosaki University Graduate School of Medicine, Hirosaki, 036-8562 Japan; 4 Department of Pathology, Brain Research Institute, Niigata University, Niigata, 951-8585 Japan; 5 Laboratory of Pharmacology and Neurobiology, College of Pharmaceutical Sciences, Ritsumeikan University, Kusatsu, 525-8577 Japan; 6 Department of Neurocognitive Science, Institute of Brain Science, Nagoya City University Graduate School of Medical Sciences, Nagoya, 467-8601 Japan; 7 Department of Neurology, Biomedical Research Center, Hirosaki University Graduate School of Medicine, Hirosaki, 036-8562 Japan; 8 Queen Square Brain Bank for Neurological Disorders, UCL Queen Square Institute of Neurology, 1 Wakefield Street, London, WC1N 1PJ UK; 9 Hirosaki University Graduate School of Health Sciences, Hirosaki, 036-8564 Japan; 10 Department of Neurodegenerative Disease, UCL Queen Square Institute of Neurology, University College London, London, WC1N 3BG UK; 11 Department of Forensic Medicine, Hirosaki University Graduate School of Medicine, Hirosaki, 036-8562 Japan; 12 Department of Clinical and Movement Neurosciences, UCL Queen Square Institute of Neurology, University College London, London, WC1N 3BG UK; 13 Reta Lila Weston Institute of Neurological Studies, UCL Queen Square Institute of Neurology, London, WC1N 3BG UK

**Keywords:** α-Synuclein, Extracellular vesicles, SNARE complex, Synucleinopathy, SYT13

## Abstract

**Background:**

Despite increasing in vitro research, direct evidence of how abnormal α-synuclein (α-Syn) dysregulates vesicular transport and synaptic function in the human brain is lacking.

**Methods:**

We performed a transcriptome analysis using brain tissues from a multiple system atrophy (MSA) mouse model, which develops human α-Syn-positive glial cytoplasmic inclusion-like structures and neuronal cytoplasmic inclusion-like structures after tamoxifen injection. We then performed histologic and biochemical analyses using brain samples from 71 human cases (Parkinson’s disease, *n* = 10; dementia with Lewy bodies [DLB], *n* = 19; MSA, *n* = 15; control: *n* = 27), a human blood sample (control: *n* = 1), and cultured cells.

**Results:**

Based on the transcriptome of the MSA mouse model, we identified 10 vesicular transport proteins, including synaptotagmin 13 (SYT13), that might interact with α-Syn. Immunohistochemistry using human brain samples demonstrated that of the 10 vesicular transport proteins identified in the transcriptome analysis, only SYT13 was incorporated into both Lewy bodies and glial cytoplasmic inclusions. Proximity ligation assays revealed that SYT13 exhibited a higher degree of interactions with phosphorylated α-Syn than with endogenous α-Syn. Immunoprecipitation confirmed that SYT13 bound predominantly to phosphorylated α-Syn, SYT1, and the soluble N-ethylmaleimide-sensitive attachment protein receptor (SNARE) complexes. Filter trap assays revealed interactions between SYT13 and soluble toxic β-sheet-rich α-Syn oligomers. Furthermore, fraction analysis showed a significant increase of SYT13 protein levels at the synapses in DLB and MSA. Notably, a correlation was observed between the levels of SYT13 and aggregated α-Syn at the synapses. SYT13 was observed to regulate extracellular vesicle release in association with SYT1 and the SNARE complexes in SH-SY5Y cells. SYT13 overexpression in SH-SY5Y cells impaired extracellular vesicle release. Consistently, the numbers of extracellular vesicles were significantly reduced in the brain homogenates of DLB and MSA cases compared with those in controls.

**Conclusions:**

Abnormal α-Syn impairs extracellular vesicle release through interactions with SYT13 in synucleinopathies. Our findings provide insights into therapeutic strategies for alleviating dysregulations of vesicular transport and synaptic function in patients with synucleinopathies.

**Supplementary Information:**

The online version contains supplementary material available at 10.1186/s40035-025-00493-6.

## Background

Abnormal α-synuclein (α-Syn) accumulates in the brains of individuals with synucleinopathies, including Lewy body diseases (Parkinson’s disease [PD] and dementia with Lewy bodies [DLB]) and multiple system atrophy (MSA) [1–3]. This causes neurodegeneration over time, which ultimately results in the loss of neurons in the affected regions. However, cells can exhibit varying degrees of resilience or susceptibility to abnormal α-Syn, depending on their intrinsic biological properties and/or the external milieu [4, 5]. This could enable certain regions of neurons to survive for longer periods, despite being unable to function physiologically. Up to 37% of patients with pathologically proven MSA develop mild-to-moderate cognitive impairment during life [6, 7]. Notably, toxic β-sheet-rich α-Syn oligomers cause synaptic dysfunction without neuronal loss and are the pathological substrate associated with cognitive impairment in MSA [7, 8]. Importantly, intranasal administration of trehalose reduces toxic α-Syn oligomers and improves memory in the MSA model [9]. Lewy bodies, the pathological hallmark of Lewy body diseases, are frequently and widely observed in the cerebral cortexes of patients with DLB who develop fluctuating cognitive impairment [10, 11]. However, fluctuations in cognitive impairment cannot be explained on the basis of neuronal loss alone. Indeed, cortical atrophy in DLB is typically less severe than that observed in Alzheimer’s disease. Lewy body formation is not correlated with neuronal loss in the high-order association cortex [12]. Of note, no neuronal loss is observed in the same region unless there is concomitant Alzheimer’s disease pathology [12]. Therefore, cognitive impairment in synucleinopathies could be attributed to various degenerative changes, including synaptic dysfunction caused by aberrant α-Syn oligomers.

Synaptic dysfunction represents a pivotal pathological feature of synucleinopathies [13–15]. Physiologically, α-Syn, which exists predominantly in a monomeric form in presynaptic nerve terminals of the human brain, plays a role in vesicular transport activities by regulating synaptic vesicle recycling, exocytotic fusion pore dilation, and the assembly of soluble N-ethylmaleimide-sensitive attachment protein receptors (SNAREs) [16–18]. However, under pathological conditions, α-Syn monomers are converted to toxic β-sheet-rich oligomers and then into aggregates, including protofibrils or fibrils [19, 20]. The aggregation of abnormal α-Syn has been speculated to lead to the loss of α-Syn functions, via the recruitment of physiologically active α-Syn, as well as neighbouring synaptic proteins, from their original positions to pathological α-Syn. Furthermore, our previous reports showed that immunoreactivity for vesicle-associated membrane protein binding protein B (VAPB), which tethers the endoplasmic reticulum to intracellular organelles, is diminished in neurons containing aberrant α-Syn in Lewy body diseases and MSA [21, 22]. Numerous vesicular structures labelled with antibodies against α-Syn and VAPB are observed in the granulofilamentous structures within the cytoplasm and nucleus of both oligodendrocytes and neurons of patients with MSA [21]. These may affect various vesicular transport activities including exocytosis at the synapses. Indeed, increasing in vitro research suggests that abnormal α-Syn has detrimental effects on synaptic functions, by not only sequestering newly produced physiological α-Syn monomers [23, 24] but disrupting SNARE complex formation or SNARE-mediated vesicle fusion, which is essential for exocytosis [16, 25–28]. However, some results appear contradictory, as many of these studies have been performed in vitro under non-physiological conditions [26–28]. Therefore, direct evidence on how abnormal α-Syn dysregulates vesicular transport activities including exocytosis at the synapses in the human brain is still lacking.

To address this knowledge gap, we first performed a whole-brain transcriptome analysis based on an adult-onset mouse model of MSA, in which inducible human α-Syn is expressed in oligodendrocytes using a Cre-loxP system [29]. This model demonstrates the formation of copious human α-Syn-positive glial cytoplasmic inclusion (GCI)-like structures in the brain. Furthermore, to a lesser extent, human α-Syn-positive neuronal cytoplasmic inclusion (NCI)-like structures are also found over time despite the absence of human α-Syn mRNA in neurons shortly following human α-Syn induction, suggesting the propagation of human α-Syn between cells [29]. This allowed for the identification of 10 vesicular transport proteins, including synaptotagmin 13 (SYT13), a synaptic protein of unknown function, which was suggested to interact with human α-Syn. Using 71 human brain samples, one human blood sample and cultured cells, we further demonstrate that SYT13 regulates extracellular vesicle release. In addition, abnormal α-Syn interacted with SYT13 at the synapse, which led to the dysregulation of the release of extracellular vesicles in synucleinopathies.

## Materials and methods

### MSA mouse model

The MSA mouse model was generated by crossing human α-Syn-flox transgenic mice with the proteolipid protein-Cre recombinase/oestrogen receptor transgenic mice [29]. Tamoxifen (100 mg/kg, intraperitoneally) was injected once a day for five days to induce human α-Syn expression in oligodendrocytes in the MSA mouse model, as reported previously [46]. We examined whether tamoxifen injection induced human α-Syn expression in the oligodendrocytes of a human α-Syn-flox transgenic mouse (30 weeks old, male), a proteolipid protein-Cre recombinase/oestrogen receptor transgenic mouse (25 weeks old, male), or an MSA mouse model (18 weeks old, male). Furthermore, we performed BaseScope assays to verify the expression of human α-Syn mRNA in oligodendrocytes rather than in neurons of the MSA mouse model (*n* = 1, 43 weeks old, male). Subsequently, the formation of GCI- and NCI-like structures was observed in a chronological sequence (*n* = 3; 30 weeks for day 0, 17 weeks for day 7, and 18 weeks for day 30; male). In addition, RNA was isolated from the right cerebral hemisphere of the MSA mouse model (*n* = 3, 10 days after tamoxifen injection, 12 weeks old, male), or the proteolipid protein-Cre recombinase/oestrogen receptor transgenic mice or α-Syn-flox mice as a control (*n* = 3, 10 days after tamoxifen injection, 12 weeks old, male). We then performed transcriptome-wide analysis by microarray (Clariom D assay, mouse, Thermo Fisher Scientific, Waltham, MA). All animals were housed in temperature- and humidity-controlled rooms under a 12 h:12 h light:dark cycle, with illumination from 7:00 a.m. to 7:00 p.m. Mice were housed 3–5 per cage, and food and water were provided ad libitum. All pertinent information regarding the methodology is provided in Additional file 1.

### Cell culture and gene constructs

The HEK293 and SH-SY5Y cells were obtained from the Japanese Collection of Research Bioresources Cell Bank (Osaka, Japan) and American Type Culture Collection (Manassas, VA), respectively. SH-SY5Y cells stably expressing human α-Syn were generated as reported previously [30]. Full-length Flag-tagged human SYT13 (OL01313882APP, Amerigo Scientific, Central Islip, NY), deletion mutants of Flag-tagged human SYT13 (Δ1-159, C2A and C2B) and human α-Syn cDNAs were prepared. The base sequences of flag-tagged human SYT13 (Δ1-159, C2A and C2B) were determined on the basis of the following references: https://www.ncbi.nlm.nih.gov/protein/NP_065877.1 and https://www.ncbi.nlm.nih.gov/nuccore/NM_020826.3. Serine residue at position 129 was substituted with glutamic acid (E) to create a phosphorylation-mimic mutant (S129E α-Syn), as reported previously [31]. For immunoprecipitation (IP), HEK293 cells were co-transfected with cDNA containing S129E α-Syn and a combination of Flag-tagged SYT13 cDNA (full-length, N-terminal, C2A or C2B). Furthermore, SH-SY5Y cells were also transfected with Silencer^®^ siRNA for SYT1 (AS02MU6X, Thermo Fisher Scientific), SYT13 (AM16708, Thermo Fisher Scientific), or mammalian uncoordinated 18–1 (Munc18-1) (AS02MN4G, Thermo Fisher Scientific), and analysed by immunoblotting. Experiments involving extracellular vesicles were conducted in accordance with the guideline [32]. Given that foetal bovine serum contains a multitude of extracellular vesicles, SH-SY5Y cells were cultured in media supplemented with extracellular vesicle-free 10% foetal bovine serum (EXO-FBSHI-50A-1, System Biosciences, Palo Alto, CA) (Fig. S1a). Extracellular vesicles were then isolated from the culture supernatant of SH-SY5Y cells transfected with full-length SYT13 cDNA, siRNA SYT13, siRNA SYT1 or siRNA control, using magnetic beads with Tim4, which specifically binds to phosphatidylserine on the surface of extracellular vesicles (MagCapture™ Exosome Isolation Kit, 290-84103, FUJIFILM Wako, Osaka, Japan). The presence of extracellular vesicles in the culture supernatant of SH-SY5Y cells was confirmed by immunoblotting using three extracellular vesicle-specific antibodies CD81 (Monoclonal/B-11, sc-166029, Santa Cruz Biotechnology, Dallas, TX), CD9 (4H7B9, 60232-1-Ig, Proteintech, Rosemont, IL), and CD63 (25682-1-AP, Proteintech), and transmission electron microscopy (Fig. S1b–d). Cell lysates were subjected to immunoblotting analysis. All pertinent information regarding the gene constructs and methodology is provided in Additional file 1.

### Human cases

Autopsy cases were provided by Hirosaki University and Niigata University. All analyses using human samples were approved by the Institutional Ethics Committee of Hirosaki University Graduate School of Medicine (No. 2023–018). A total of 72 autopsy cases were examined for a range of analyses (immunohistochemistry, immunoblotting, proximity ligation assay [PLA], IP, fraction analysis, filter trap assay, and RNAscope analysis). These cases included brains of patients with PD (*n* = 10), DLB (*n* = 19), and MSA (*n* = 15), as well as normal controls (*n* = 27), and one blood sample (control: *n* = 1). A number of cases were subjected to multiple analyses. For each analysis, the sex, age and postmortem delay of the subjects were matched between the groups. Furthermore, the fixation time was also matched between the groups for immunohistochemistry, PLA, and RNAscope analysis. Experiments involving extracellular vesicles were conducted in accordance with the guideline [32]. Extracellular vesicles were isolated using MagCapture™ Exosome Isolation Kit. Extracellular vesicles isolated from the brain homogenate were confirmed by immunoblotting using three extracellular vesicle-specific antibodies (CD81, CD9, and CD63), transmission electron microscopy, and interferometric light microscopy (Fig. S1b, e–g). Demographic data for the human cases are presented in Additional file 3: Table S1. All pertinent information regarding the methodology was provided in Additional file 1.

### Analyses

All methods in the present study are detailed in Additional file 1; these include transcriptome analysis by microarray, immunohistochemistry, double immunofluorescent staining, AlphaFold 3 analysis, PLA, immunoblotting, IP, BaseScope assay, RNAscope assay, fraction analysis, as well as semi-quantitative and quantitative analysis. Out of the three rabbit anti-SYT13 antibodies (NBP2-93419, Novus Biologicals, Centennial, CO; MBS9606007, MyBioSource, San Diego, CA; ABIN2840354, Antibodies-online.com, Limerick, PA) (Fig. S2), MBS9606007 was selected for IP using human brain tissues. Information regarding all antibodies used in the present study is included in Additional file 4: Table S2.

### Statistical analysis

All statistical analyses in the present study were performed using SPSS 26.0 (SPSS Inc., Chicago, IL). To compare two groups, either a* t*-test for parametric data or a Mann − Whitney U test for non-parametric data was performed, based on the normality of the data evaluated by the Shapiro − Wilk test. For the comparison of more than two groups, the Shapiro–Wilk test was initially performed, followed by one-way analysis of variance with Tukey for parametric data. When data exhibited a normal distribution, a column graph was used to illustrate the mean and standard deviation. When data were not normally distributed, they were presented with a box-and-whisker plot. *P* < 0.05 was considered to be statistically significant.

## Results

### Identification of transcripts of 10 vesicular transport proteins in the MSA mouse model

**Fig. 1:**
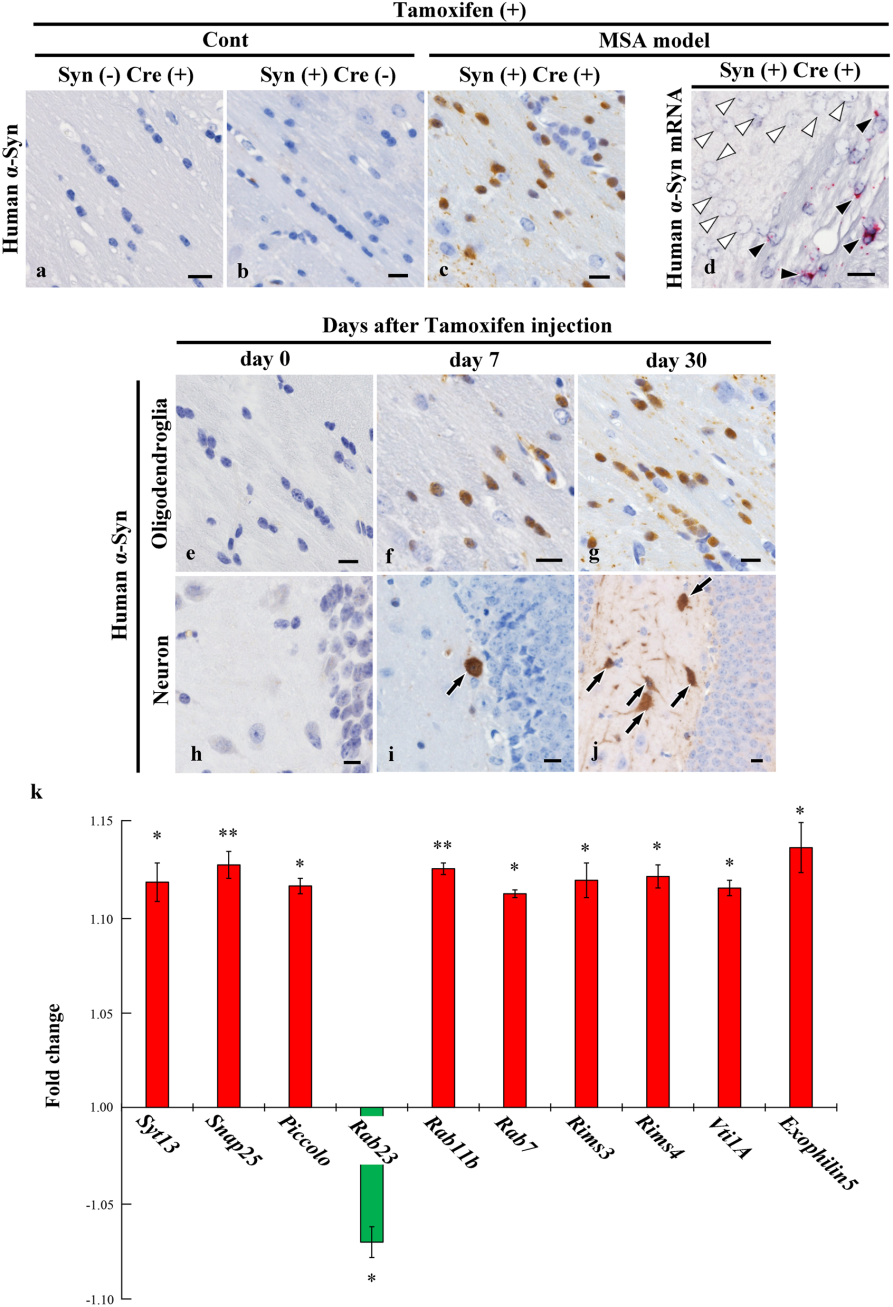
Transcripts of 10 vesicular transport proteins in a multiple system atrophy (MSA) mouse model. **a**, **b** Intraperitoneal injection of tamoxifen did not induce human α-synuclein (α-Syn) expression in control mice (α-synuclein-flox transgenic or the proteolipid protein-Cre recombinase/oestrogen receptor transgenic mouse). **c** Human α-Syn expression was induced in the oligodendrocytes of a mouse model of MSA after injection of tamoxifen. **d** Human *α-Syn* mRNA expression detected in oligodendrocytes in the corpus callosum after tamoxifen injection (black arrowheads), but not in neurons in the cerebral cortex (white arrowheads). **e**–**j** Time course analysis of human α-Syn expression in oligodendrocytes and neurons in the MSA mouse model. Human α-Syn was not expressed in oligodendrocytes and neurons immediately before tamoxifen injection (day 0) (**e**, **h**). Many human α-Syn-positive glial cytoplasmic inclusion (GCI)-like structures and a few human α-Syn-positive neuronal cytoplasmic inclusion (NCI)-like structures were formed seven days after tamoxifen injection (**f**, **i**). GCI-like and NCI-like structures were increased at 30 days after tamoxifen induction (**g**, **j**). **k** Transcripts of 10 vesicular transport proteins were significantly altered in the MSA mouse model 10 days after induction of human α-Syn. **a**–**j** Human α-Syn (syn211). Scale bars, 10 μm. Unpaired one-way analysis of variance was performed to compare gene expression between control and the MSA mouse model. *P < 0.05, **P < 0.01

In control mice (α-Syn-flox transgenic or proteolipid protein-Cre recombinase/oestrogen receptor transgenic mice), we confirmed that human α-Syn expression was not induced after tamoxifen injection (Fig. 1a, b). However, this injection successfully induced human α-Syn expression in oligodendrocytes of the MSA model mice (Fig. 1c). In addition, the presence of human α-Syn mRNA was confirmed in oligodendrocytes, but not in neurons, of the MSA mouse model (Fig. 1d). Subsequently, the chronological formation of GCI- and NCI-like structures in the MSA mouse model was examined. Results demonstrated the formation of multiple GCI-like structures seven days after human α-Syn induction, and the formation of NCI-like structures was also observed to a lesser extent (Fig. 1e–j), consistent with previous report [8]. These findings suggest the propagation of human α-Syn between cells. To identify vesicular transport proteins that potentially interact with α-Syn, we then performed a transcriptome analysis of the right cerebral hemisphere of MSA (*n* = 3) and control (*n* = 3) mice. Of the 6878 differentially expressed transcripts, 10 mRNAs encoding vesicular transport proteins were identified, including *Syt13*, synaptosomal-associated protein 25 kDa (*Snap25*), *Piccolo*, Ras-related protein *Rab23*, *Rab11b*, *Rab7*, regulating synaptic membrane exocytosis (*Rims*) 3, *Rims4*, vesicle transport through interaction with T-SNAREs 1A (*Vti1A*), and *Exophilin5* (Fig. 1k). Of the 17 Syt isoforms, only *Syt13* mRNA level was significantly changed after human α-Syn induction. The functions of these proteins related to vesicular transport and locations, are summarised in Additional file 5: Table S3 [33–46]. The top 10 upregulated and downregulated genes are shown in Table S4. The raw microarray data were also deposited in the GEO database (http://www.ncbi.nlm.nih.gov/geo) under the accession number GSE129531.

### SYT13 is incorporated into Lewy bodies and GCIs

**Fig. 2:**
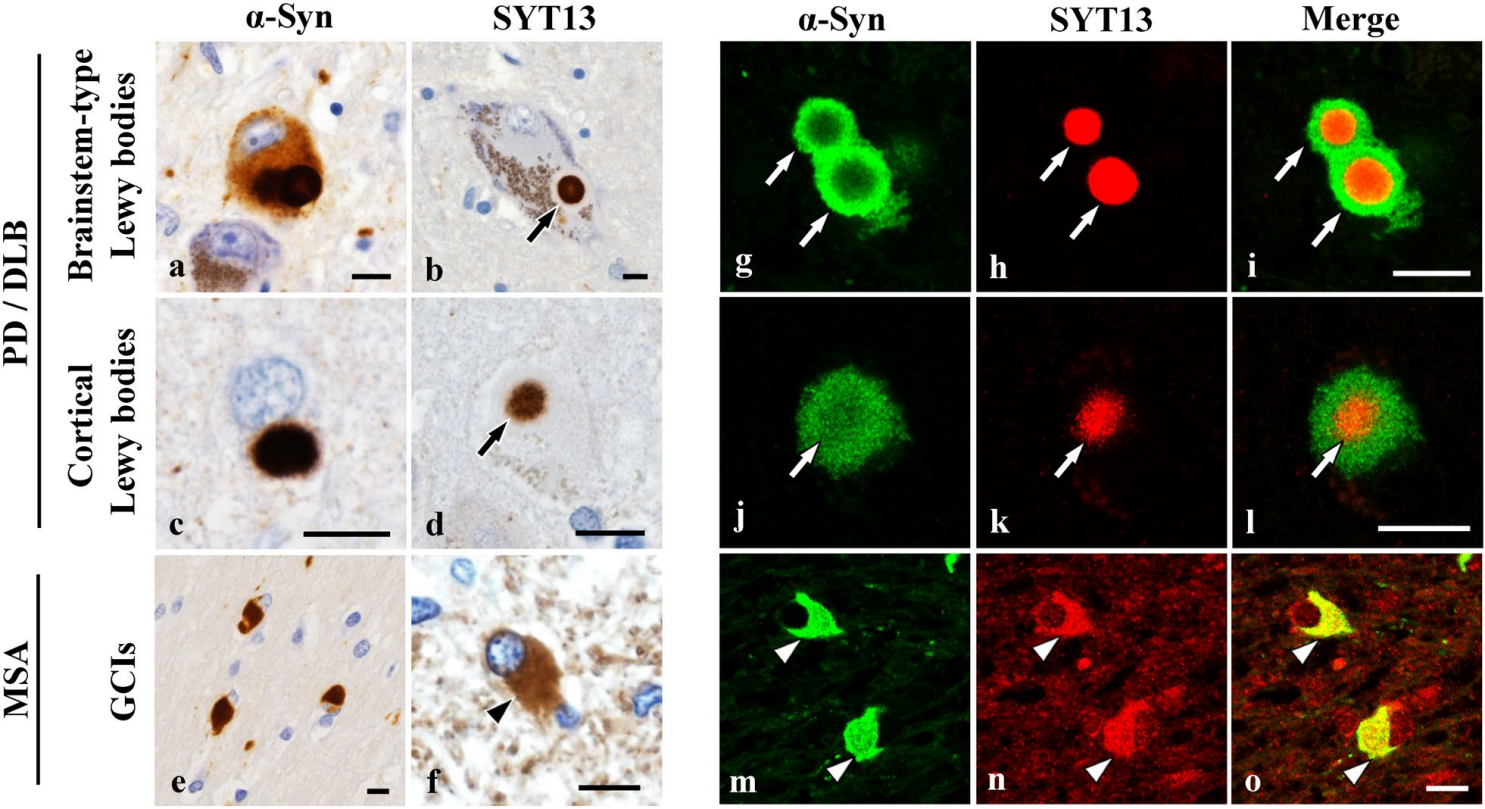
Incorporation of SYT13 into Lewy bodies and glial cytoplasmic inclusions (GCIs). **a**–**f** Immunohistochemical analysis of the human brain tissues of patients with Parkinson’s disease (PD) (*n* = 7), dementia with Lewy bodies (DLB) (*n* = 7), and multiple system atrophy (MSA) (*n* = 7), and controls (*n* = 7). Lewy bodies and GCIs were immunopositive for α-Syn (syn211). Immunohistochemistry shows the incorporation of SYT13 into both Lewy bodies (**b**, **d**, arrows) and GCIs (**f**, arrowhead). **g**–**o** Double immunofluorescence staining confirmed the presence of SYT13 in Lewy bodies (**g**–**l**, white arrows) and GCIs (**m**–**o**, white arrowheads). Scale bars, 10 μm

**Fig. 3:**
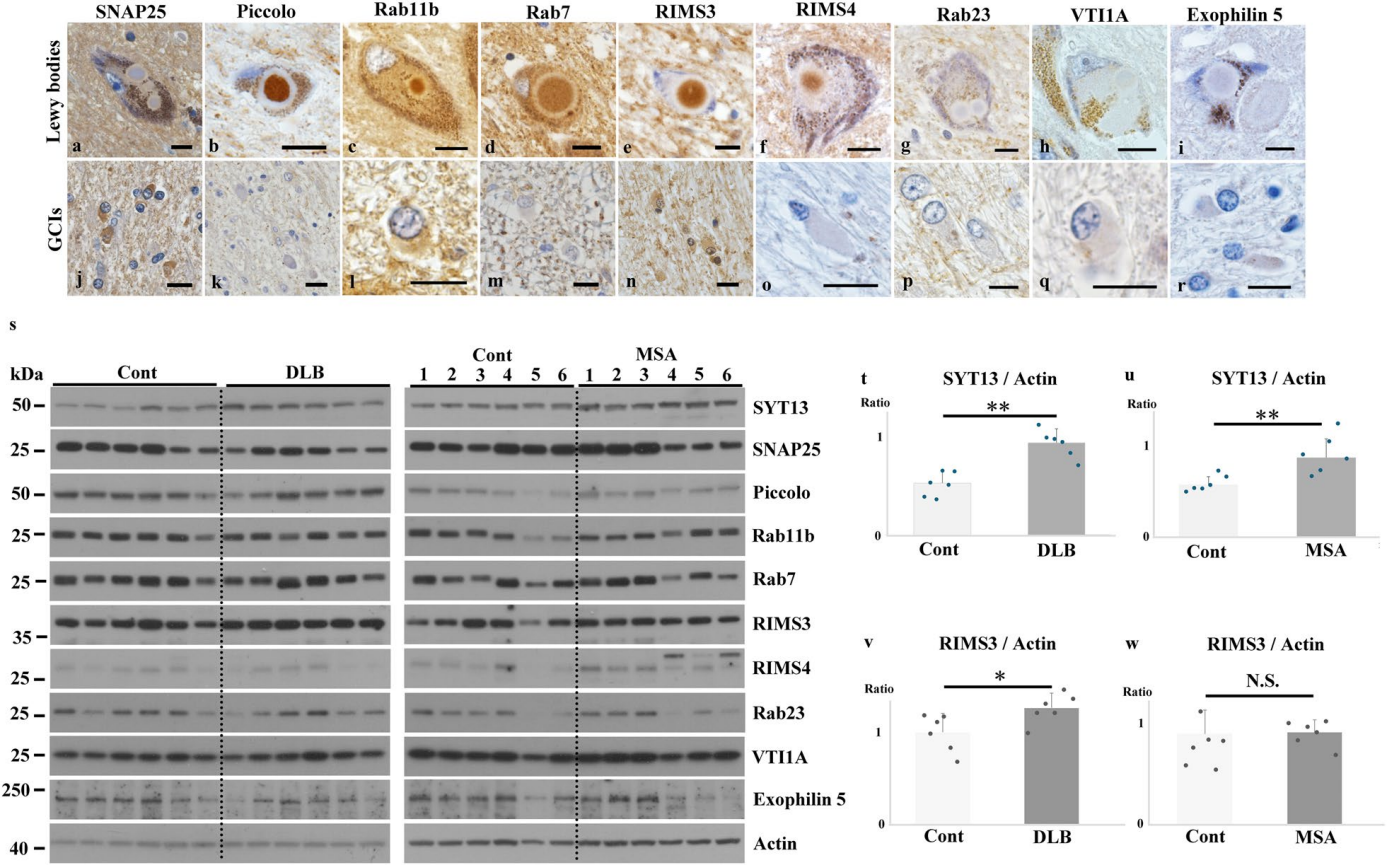
Alterations of 10 vesicular transport proteins in Lewy body diseases and multiple system atrophy (MSA). **a**–**r** Immunohistochemical analysis of human brain sections using antibodies for 9 vesicle transport proteins identified by the transcriptome analysis in the mouse model of MSA. Brain tissues of patients with Parkinson’s disease (*n* = 7), dementia with Lewy bodies (DLB) (*n* = 7) and MSA (*n* = 7), and controls (*n* = 7) were used for this analysis. SNAP25 was incorporated into glial cytoplasmic inclusions (GCIs) but not into Lewy bodies (**a**, **j**). Lewy bodies were immunopositive for Piccolo (**b**), Rab11b (**c**), Rab7(**d**), RIMS3 (**e**) and RIMS4 (**f**) but not GCIs (**k**–**o**). Rab23, VTI1A and Exophilin5 were not incorporated into both Lewy bodies and GCIs (**g**–**i**, **p**–**r**). **s** Immunoblotting using temporal lobe tissues of human cases (control: *n* = 6 and DLB: *n* = 6; control: *n* = 6 and MSA: *n* = 6) for the 10 vesicle transport proteins. **t**–**w** Elevated SYT13 protein levels were observed in patients with DLB (**t**) and MSA (**u**), compared with the control groups, whereas RIMS3 protein level was elevated only in patients with DLB (**v**, **w**). Scale bars, 10 μm

Next, we examined whether the 10 vesicular transport proteins identified in the transcriptome analysis are involved in Lewy bodies and GCIs, the defining cytopathological hallmarks of Lewy body diseases and MSA, respectively. To this end, we performed immunohistochemistry using human brains (PD: *n* = 7; DLB: *n* = 7; MSA: *n* = 7). Only SYT13 was incorporated into both α-Syn-positive Lewy bodies and GCIs (Fig. 2a–f). Moreover, double immunofluorescence staining confirmed the presence of SYT13 in Lewy bodies and GCIs (Fig. 2g–o). The SYT13 positive rates in brainstem-type Lewy bodies and GCIs in serial sections were 100% (91/91) and 56% (140/250), respectively. While GCIs were immunopositive for SNAP25, no Lewy bodies stained positive when using an anti-SNAP25 antibody (Fig. 3a, j). Conversely, Lewy bodies, but not GCIs, were immunopositive for Piccolo, Rab11b, Rab7, RIMS3 and RIMS4 (Fig. 3b–f, k–o). The other proteins (Rab23, VTI1A, and Exophilin5) were not incorporated into Lewy bodies or GCIs (Fig. 3g–i, p–r). We further performed immunoblotting on the human brains [control (*n* = 6) and DLB (*n* = 6); control (*n* = 6) and MSA (*n* = 6)] using antibodies against SYT13, SNAP25, Piccolo, Rab23, Rab11b, Rab7, Rims3, Rims4, VTI1A and Exophilin5 (Fig. 3s). Consistent with the findings of immunohistochemistry (Fig. 2b, d, f), we found significantly higher protein levels of SYT13 in DLB and MSA than in the control groups (Fig. 3t, u). Compared with the control groups, the RIMS3 levels were elevated in patients with DLB alone (Fig. 3v, w). No differences in protein levels of the other proteins were seen between control cases and patients with DLB or MSA (Fig. 3s). Therefore, we focused on SYT13 in the following experiments.

### SYT13 interacts more with phosphorylated α-Syn than with endogenous α-Syn

**Fig. 4:**
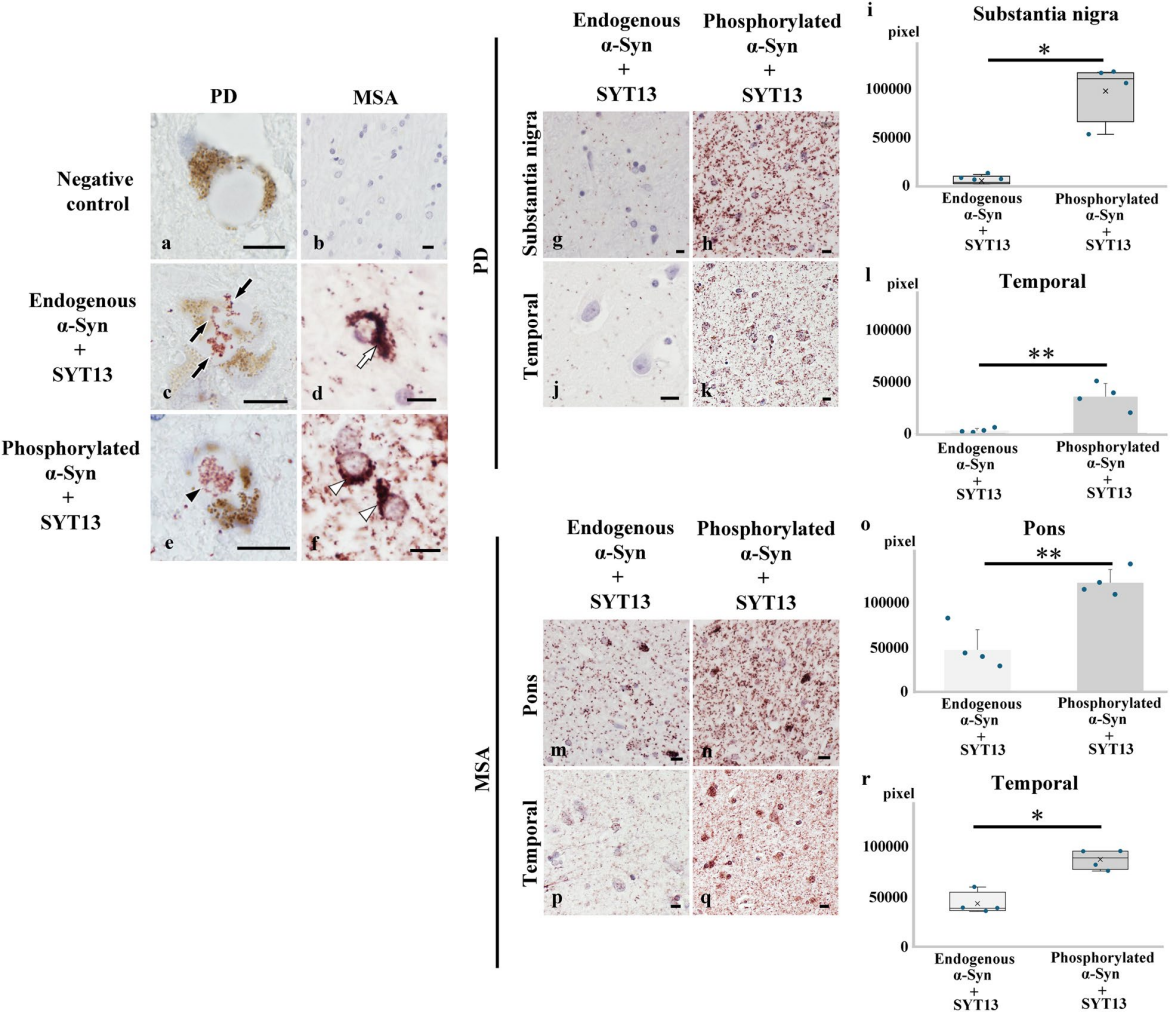
SYT13 interacts more with phosphorylated α-Syn than with endogenous α-Syn. A proximity ligation assay (PLA) was performed to visualise protein–protein interactions between SYT13 and α-Syn in the affected regions. **a**, **b** No signals were observed in Lewy bodies and glial cytoplasmic inclusions (GCIs) in the absence of primary antibodies. **c**-**f** The presence of strings of PLA signals indicates an interaction between SYT13 and both endogenous α-Syn in Lewy bodies (**c**, arrows) and a GCI (**d**, white arrow) and phosphorylated α-Syn in a Lewy body (**e**, black arrowhead) and in GCIs (**f**, white arrowheads). **g**–**r** The semi-quantification of PLA signals indicated that SYT13 interacts with phosphorylated α-Syn to a greater extent than with endogenous α-Syn in the substantia nigra (**g, h, i**) and the temporal lobe (**j, k, l**) of Parkinson’s disease (PD) cases (*n* = 4), and the pons (**m, n, o**) and the temporal lobe (**p, q, r**) of multiple system atrophy (MSA) cases (*n* = 4) compared with that observed in control cases (*n* = 4). Data in (**i, r**) are presented as a box-and-whisker plot and were analysed via a Mann–Whitney U test. Data in (**l, o**) display the mean ± SD and were analysed with two-sample *t*-test. Scale bars, 10 μm. * *P* < 0.05; ** *P* < 0.01

The results from transcriptomic, immunohistochemical, and immunoblotting analyses indicated the involvement of SYT13 in the pathogenesis of synucleinopathies. As α-Syn is an intrinsically disordered synaptic protein that frequently interacts with other proteins under pathological conditions [21, 22], we speculated that abnormal α-Syn may interact with SYT13 in synucleinopathies. Therefore, we performed PLA, which visualises protein–protein interactions [8, 31], using the human brains [PD (*n* = 4) and control (*n* = 4); MSA (*n* = 4) and control (*n* = 4)]. No signals were found without the use of primary antibodies (Fig. 4a, b). However, we found strings of signals indicating interactions between SYT13 and endogenous as well as phosphorylated α-Syn in Lewy bodies and GCIs (Fig. 4c–f). We then semi-quantified the interaction between SYT13 and endogenous or phosphorylated α-Syn (Fig. 4g–r); this showed greater interaction between SYT13 and phosphorylated α-Syn than that between SYT13 and endogenous α-Syn in the substantia nigra and the temporal cortex of patients with PD (Fig. 4g–l), and in the pontine base and the temporal cortex of patients with MSA (Fig. 4m–r)

**Fig. 5:**
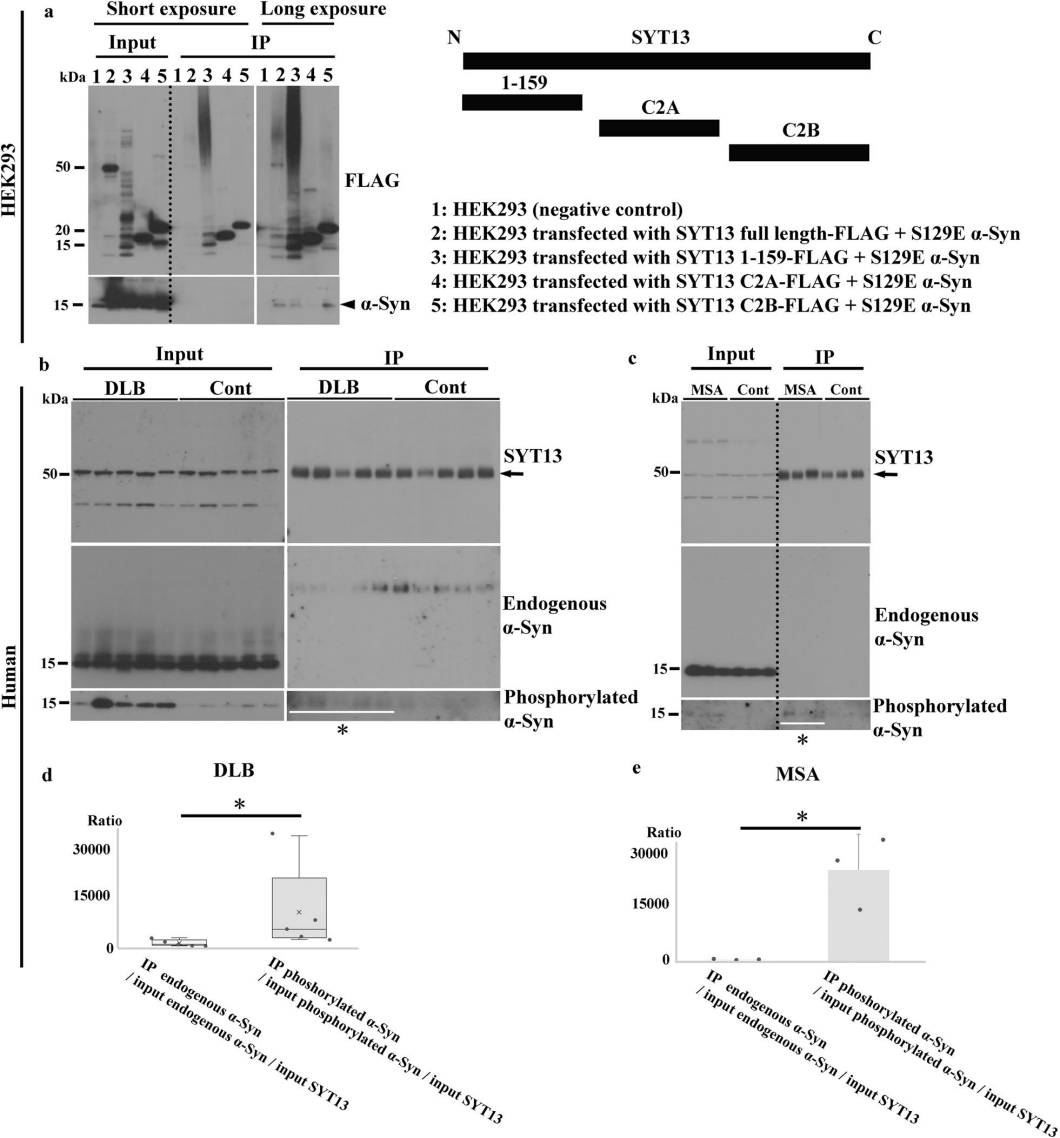
SYT13 binds to abnormal α-Syn. **a** FLAG-tagged SYT13 was immunoprecipitated from the cell lysates of HEK293 cells, as a negative control (lane 1), and HEK293 cells co-transfected with one of the SYT13 constructs (lane 2, full-length; lane 3, Δ1-159; lane 4, C2A; or lane 5, C2B) and S129E α-Syn, a phosphorylation-mimic mutant in which serine residue (S) at position 129 was substituted with glutamic acid (E). Anti-FLAG antibody was used for this analysis. The expression levels of α-Syn and SYT13 were analysed via immunoblotting. In addition to full-length SYT13, the co-immunoprecipitation of α-Syn with the C2B domain of SYT13 was detected (arrowhead). **b, c** To compare the interaction between SYT13 and endogenous versus phosphorylated α-Syn, temporal lobe homogenates of dementia with Lewy bodies (DLB) (*n* = 5) and control cases (*n* = 5) as well as multiple system atrophy (MSA) (*n* = 3) and control cases (*n* = 3) were immunoprecipitated using an antibody against SYT13 (arrows). No bands indicating the binding of endogenous α-Syn and SYT13 were observed in the controls and samples from the DLB and MSA cases, whereas SYT13 was found to bind phosphorylated α-Syn in all DLB and MSA cases (asterisks). **d, e** In patients with DLB and MSA, the higher interaction between phosphorylated α-Syn and SYT13 was observed compared with endogenous α-Syn and SYT13. The Y-axis indicates the extent of binding of SYT13 to endogenous or phosphorylated α-Syn.

We then immunoprecipitated SYT13 from the cell lysates of HEK293 cells co-transfected with one of the *SYT13* gene constructs (full-length, Δ1-159, C2A, or C2B) and S129E α-Syn, and examined the lysates through immunoblotting. S129E α-Syn was co-immunoprecipitated with full-length SYT13 and the C2B domain (Fig. 5a). To compare the interaction between SYT13 and endogenous versus phosphorylated α-Syn, we also immunoprecipitated SYT13 from the human temporal lobe homogenates (DLB *n* = 5 and control *n* = 5; MSA *n* = 3 and control *n* = 3). No bands representative of endogenous α-Syn were co-immunoprecipitated with SYT13 (Fig. 5b, c), whereas distinct bands corresponding to phosphorylated α-Syn were found in DLB and MSA cases (Fig. 5b, c, asterisks). In patients with DLB and MSA, the higher interaction of phosphorylated α-Syn and SYT13 was observed compared with endogenous α-Syn and SYT13 (Fig. 5d, e). Moreover, we predicted the folded conformation and protein–protein interaction of α-Syn and SYT13 by AlphaFold 3. Results suggested a higher degree of interaction between S129E α-Syn and SYT13 C2B. The distances between S129E α-Syn T33 and SYT13 C2B Q417 and between α-Syn Y39 and SYT13 C2B Q419 were 2.9 Å and 3.2 Å, respectively. However, the interaction between SYT13 and wild-type α-Syn was comparatively weaker. The distances between wild-type α-Syn T33 and SYT13 C2B Q417 and between α-Syn Y39 and SYT13 C2B Q419 were 16.2 Å and 3.6 Å, respectively (Fig. S3a–d and Supplementary movie).

### SYT13 interacts with toxic β-sheet-rich α-Syn oligomers in synucleinopathies

**Fig. 6:**
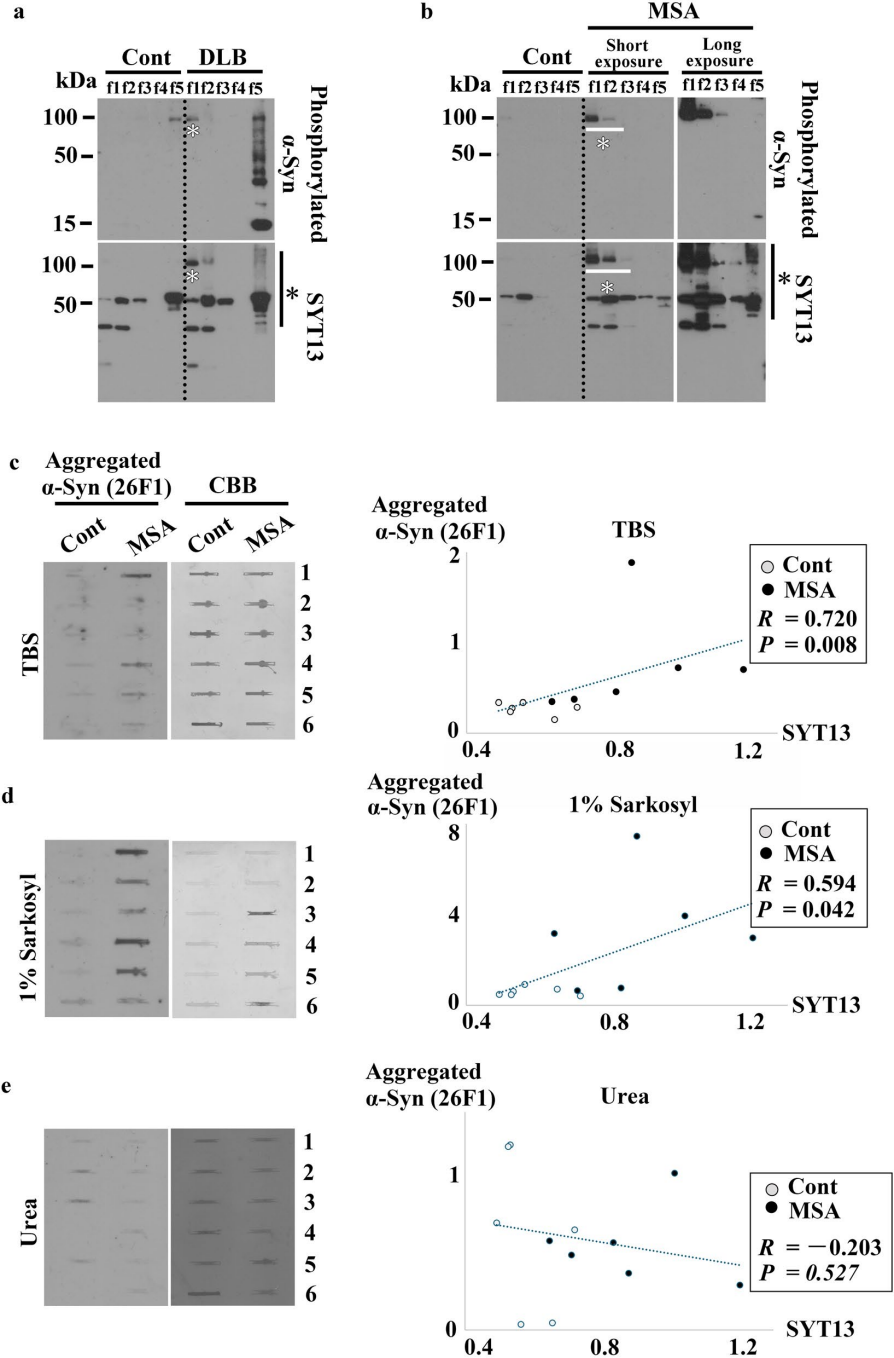
SYT13 interacts with toxic β-sheet-richα-Syn oligomers in synucleinopathies. **a**, **b** To qualitatively investigate the changes in SYT13 in synucleinopathies, we performed fraction analysis (DLB, *n* = 3 and control *n* = 3; MSA, *n* = 3 and control *n* = 3) using the human temporal lobe homogenates. Representative data demonstrate the presence of SYT13 smear bands in the urea-insoluble fraction (f5) of DLB (**a**) and MSA (**b**) samples (black asterisks), but not in controls. In addition, the SYT13 bands were observed in the Tris-buffered saline (TBS) (f1)-soluble fraction of DLB (**a**, white asterisk) as well as the TBS (f1)- and Triton (f2)-soluble fractions of MSA samples (**b**, white asterisk). The immunoblotting patterns observed in the other DLB and MSA cases were comparable. F3, 1% sarkosyl fraction; f4, CHAPS fraction. **c**–**e** To ascertain whether the abnormal α-Syn that binds to SYT13 is predominantly composed of soluble toxic α-Syn oligomers or insoluble α-Syn fibrils, a filter trap assay was performed based on the same cases used for immunoblotting (MSA *n* = 6 and control *n* = 6) in Fig. 3s, with corresponding case numbers. The 26F1 antibody detects toxic β-sheet-rich α-Syn oligomers and fibrils. (**c**) There was a strong correlation between the protein level of SYT13 determined via immunoblotting (Fig. 3u), and the level of aggregated α-Syn in TBS (*R* = 0.720, *P* = 0.008). (**d**) A weak but significant correlation was also observed between SYT13 and aggregated α-Syn levels in the insoluble sarkosyl fraction (*R* = 0.594, *P* = 0.042). (**e**) No such correlation was observed in the insoluble urea fraction (*R* =  − 0.203, *P* = 0.527). Correlation was analysed based on Spearman’s rank correlation coefficient (**c**, **d**) or Pearson’s correlation coefficient (**e**), based on normality examined via the Shapiro–Wilk test. The protein levels of 26F1-positive α-Syn were normalised to the Coomassie Brilliant Blue (CBB) staining

To qualitatively investigate the changes in SYT13 in synucleinopathies, we performed fraction analysis (DLB *n* = 3 and control *n* = 3; MSA *n* = 3 and control *n* = 3) using the human temporal lobe homogenates. This analysis enables differentiation of the varying solubilities of α-Syn from post-mortem human brain tissues via the sequential detergent-strength and centrifugation techniques (f1: Tris-buffered saline [TBS]; f2: Triton; f3: 1% sarkosyl; f4: CHAPS; f5: urea). Thus, soluble α-Syn strains are isolated in f1 or f2 traction, whereas insoluble α-Syn strains are isolated in f3 to f5. This analysis revealed smear bands of SYT13 in the urea-insoluble fraction (f5) of DLB and MSA cases (Fig. 6a, b, black asterisks), but not in the controls. In addition, SYT13 bands were also observed in the TBS (f1) of DLB samples (Fig. 6a, white asterisks), and in the TBS (f1)- and Triton (f2)-soluble fractions of MSA samples (Fig. 6b, white asterisks). These bands, with molecular weights of 100 kDa, largely corresponded to phosphorylated α-Syn (Fig. 6a, b, white asterisks). Thus, SYT13 can be associated with not only insoluble but also soluble abnormal α-Syn.

To ascertain whether the abnormal α-Syn that binds to SYT13 is predominantly composed of soluble toxic α-Syn oligomers or insoluble α-Syn fibrils, we performed a filter trap assay using an antibody against aggregated α-Syn (26F1), which specifically detects toxic β-sheet-rich α-Syn oligomers and fibrils in this assay [47]. For this, we used the same cases used for immunoblotting (MSA *n* = 6 and control *n* = 6). We then examined the correlation between SYT13 protein levels, as determined via immunoblotting, and those of aggregated α-Syn, as measured via the filter trap assay, in the TBS, sarkosyl, and urea fractions. A strong correlation was observed between SYT13 and aggregated α-Syn levels in the TBS-soluble fraction (*R* = 0.720, *P* = 0.008; Fig. 6c). A weak but significant correlation was also observed between SYT13 and aggregated α-Syn levels in the insoluble sarkosyl fraction (*R* = 0.594, *P* = 0.042; Fig. 6d). However, no such correlation was found in the insoluble urea fraction (*R* =  − 0.203,* P* = 0.527; Fig. 6e). An explanation is that as abnormal α-Syn insolubility increased from TBS (Fig. 6c), sarkosyl (Fig. 6d), to urea fraction (Fig. 6e), the correlation between SYT13 and aggregated α-Syn was lost in the urea fraction. Together with the findings of PLA (Fig. 4g–r), these findings suggest that SYT13 may interact with soluble, toxic β-sheet-rich α-Syn oligomers, rather than α-Syn fibrils.

### SYT13 is associated with the SNARE complex, and abnormal α-Syn dysregulates extracellular vesicle release via SYT13

**Fig. 7:**
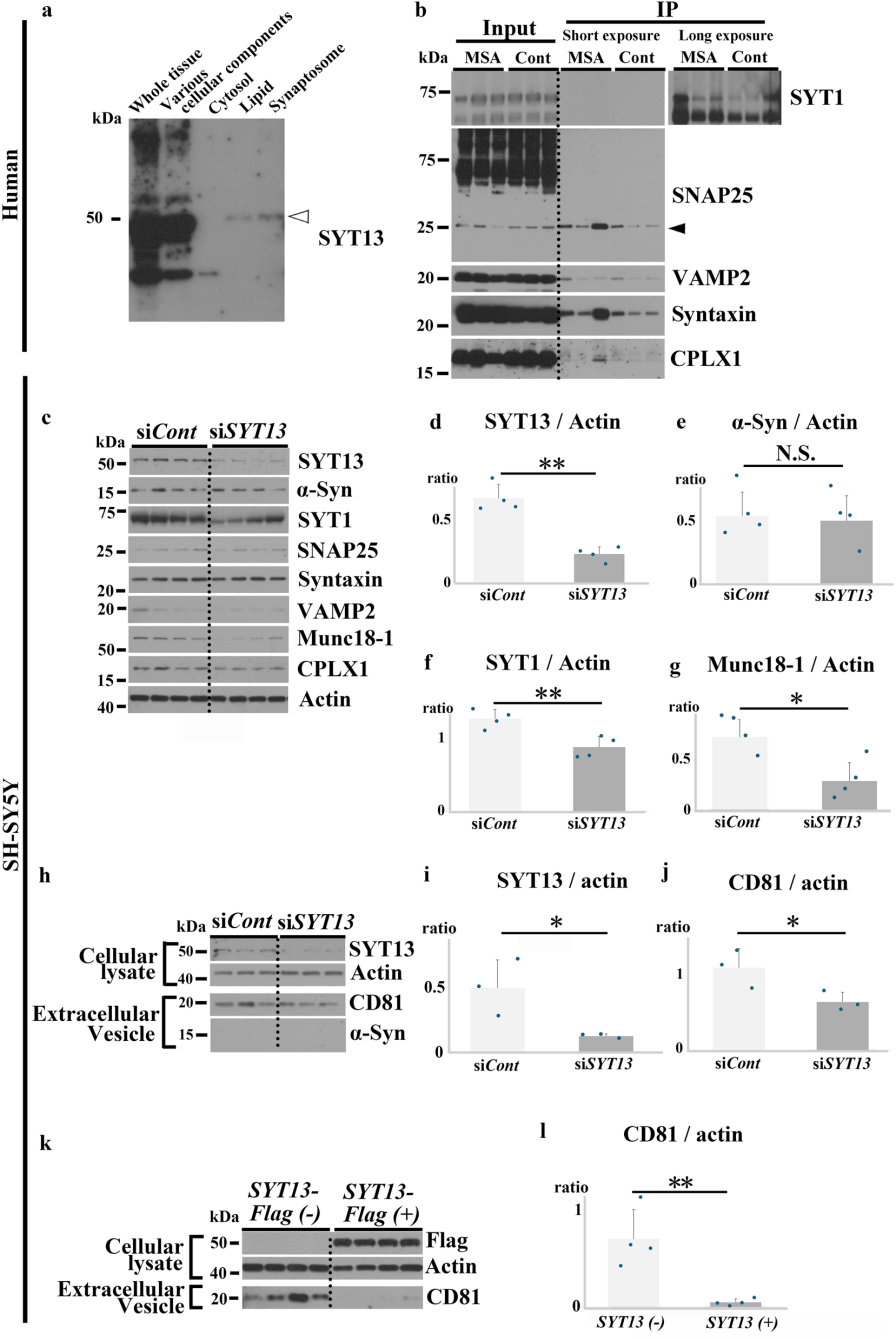
SYT13 binds to the SNARE complex and regulates extracellular vesicle release. **a** Human temporal lobes (*n* = 3) were fractionated to identify the location of SYT13 in cells. SYT13 was detected in the synaptosome fraction, but not in the cytosol fraction. **b** The temporal lobes of patients with multiple system atrophy (MSA) (*n* = 3) and controls (*n* = 3) were immunoprecipitated with an anti-SYT13 antibody. SYT13 bound SYT1, SNAP25 (arrowhead), VAMP2, Syntaxin, and CPLX1. **c**–**g** SYT13 expression was suppressed in SH-SY5Y cells to examine the interactions between SYT13 and α-Syn, SYT1, or the SNARE complexes. A significant decrease in the protein levels of SYT1 and Munc18-1 was observed in SH-SY5Y cells treated with *SYT13* siRNA compared to cells treated with control siRNA. There were no discernible differences in the protein levels of α-Syn, SNAP25, Syntaxin, VAMP2, and CPLX1 between the groups. **h**–**l** Extracellular vesicles were isolated from the culture supernatant of SH-SY5Y cells with SYT13 knockdown or overexpression. Extracellular vesicle-free 10% foetal bovine serum was used for this analysis. Both knockdown (**j**) and overexpression (**l**) of *SYT13* resulted in impaired extracellular vesicle release. No difference was observed in α-Syn protein levels in extracellular vesicles isolated from cells treated with or without *SYT13* siRNA. Mean ± SD, two-sample *t*-test. *P < 0.05; **P < 0.01

SYT1, one of the 17 SYT family proteins, possesses domains that sense Ca^2+^ at the synapses and initiates exocytosis through interactions with the SNARE complex. However, SYT13 lacks such domains [48, 49]. In addition, its location and function remain largely unknown. However, considering the similarities among the basic molecular structures of SYT family proteins and the interactions detected between SYT13 and α-Syn in the present study, we hypothesised that SYT13 may also regulate exocytosis at the synapses. For this, we fractioned the human temporal lobes to identify the location of SYT13 in cells. Results showed the presence of SYT13 in the synaptosome fraction, but not in the cytosol fraction (Fig. 7a). We then performed immunoprecipitation using the human temporal lobes (MSA *n* = 3 and control *n* = 3). As expected, in addition to α-Syn (Fig. 5b, c), SYT1 and key components of the SNARE complex, including SNAP25, VAMP2 (vesicle-associated membrane protein 2), Syntaxin, and CPLX1 (Complexin 1), were co-immunoprecipitated with SYT13 (Fig. 7b). We then knocked down SYT13 in SH-SY5Y cells to examine interactions with the SNARE complexes (Fig. 7c–g). We found a significant reduction in protein levels of SYT1 and the SNARE complex-related protein (Munc18-1) in SH-SY5Y cells treated with *SYT13* siRNA compared with those in cells treated with control siRNA (Fig. 7f, g). To confirm whether SYT13 regulates extracellular vesicle release, we isolated and quantified extracellular vesicles from the culture supernatant of SH-SY5Y cells with SYT13 knockdown or overexpression. Both SYT13 knockdown and overexpression resulted in impaired extracellular vesicle release (Fig. 7h–l). Additionally, we studied membrane capacitance of SH-SY5Y cells, which directly reflects exocytosis [50]. Consistently, knock-down and overexpression of SYT13 resulted in a significant reduction of membrane capacitance (Fig. S4a, b). To further confirm the interaction between SYT13 and SYT1 or Munc18-1, we knocked down SYT1 or Munc18-1 (Fig. S5a, b). SYT1 knockdown led to a significant reduction in the SYT13 protein levels, with a similar trend observed for Munc18-1 (*P* = 0.09) (Fig. S5c). Immunoblotting analysis of SH-SY5Y cells treated with *SYT1* siRNA revealed a significant reduction in the protein levels of Syntaxin and Munc18-1 (Fig. S5d–f). Munc18-1 knockdown also led to a reduction in Syntaxin levels (Fig. S5g, h). Additionally, extracellular vesicle release was impaired in cells treated with *SYT1* siRNA (Fig. S5i). Thus, SYT13 may regulate extracellular vesicle release in association with SYT1 and the SNARE complexes. However, α-Syn was not detected in extracellular vesicles isolated from SH-SY5Y cells treated with or without *SYT13* siRNA.

**Fig. 8:**
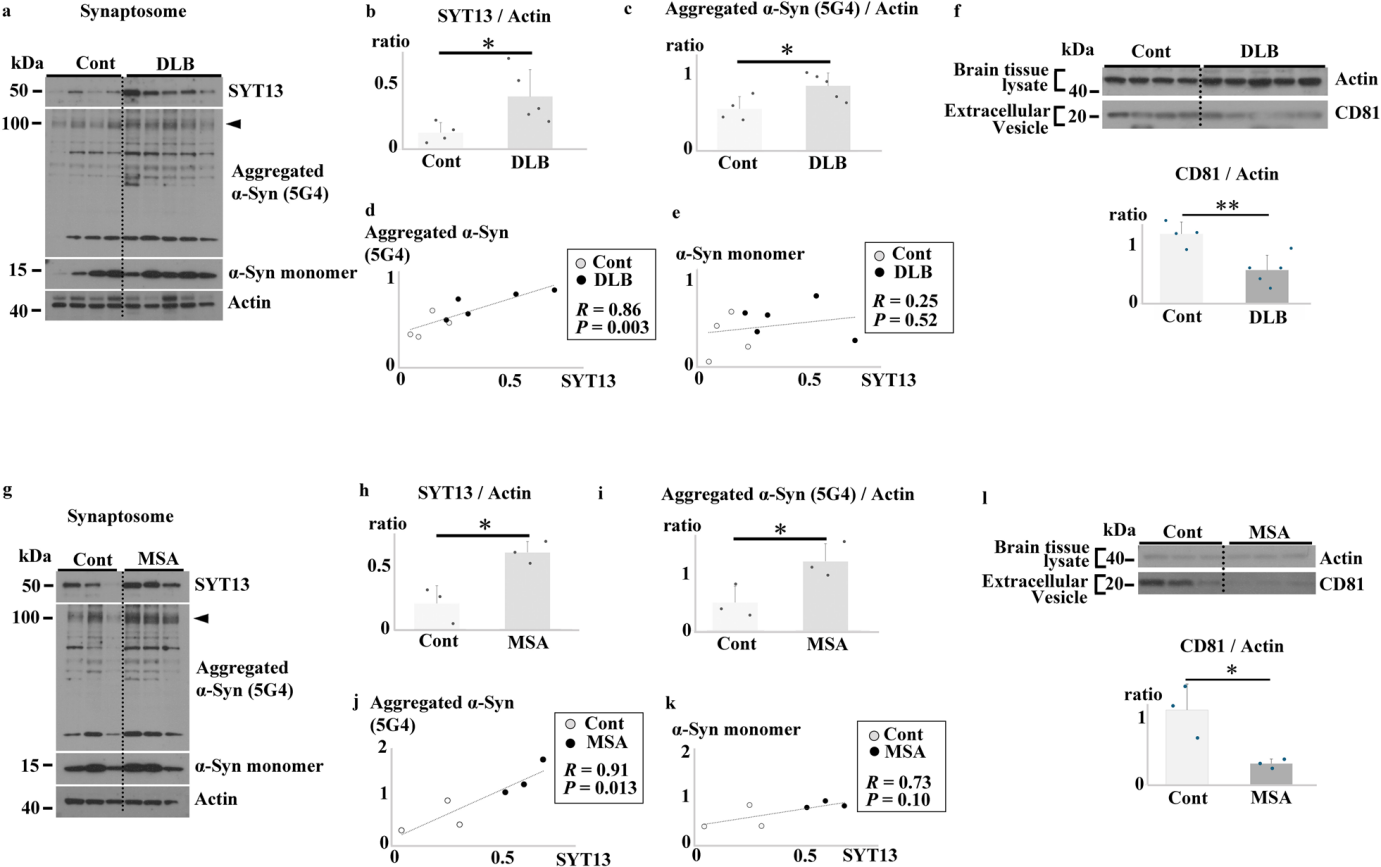
SYT13 interacts with abnormal α-Syn and disrupts extracellular vesicle release in synucleinopathies. **a**–**e**, **g**–**k** Interaction between SYT13 and α-synuclein (α-Syn) in the synaptosome fraction of human temporal lobes (DLB *n* = 5 and control *n* = 4; MSA *n* = 3 and control *n* = 3). Immunoblotting revealed a significant elevation in the SYT13 protein levels and aggregated α-Syn levels (arrowheads) in the synaptosome fraction of the brains of DLB (**b**) and MSA (**h**) cases, compared to controls. Moreover, there was a significant correlation between the protein levels of 5G4-positive aggregated α-Syn and SYT13 in DLB (*R* = 0.86, *P* = 0.003, **d**) and MSA (*R* = 0.91, *P* = 0.013, **j**). No such correlation was found between SYT13 and α-Syn monomer levels in DLB (*R* = 0.25, *P* = 0.52, **e**) and MSA (*R* = 0.73, *P* = 0.10, **k**). **f**, **l** The number of extracellular vesicles, measured using anti-CD81 antibodies, decreased significantly in the temporal lobe homogenates from patients with DLB (**f**) and MSA (**l**) compared to the controls. Data presented in (**b**,** c**,** f**,** h**,** i**, and** l**) are mean ± SD and were analysed with two-sample *t*-test. Pearson’s correlation coefficient was analysed (**d**,** e**,** j**,** k**), based on normality examined via the Shapiro–Wilk test. *P < 0.05; **P < 0.01

Because both increase and decrease of SYT13 protein level affected the extracellular vesicle release in vitro, we investigated the changes in SYT13 in the synaptosome fraction of the human brains [DLB *n* = 5 and control *n* = 4; MSA *n* = 3 and control *n* = 3] (Fig. 8a–e, g–k). Immunoblotting analysis revealed a significant elevation in SYT13 protein levels in the synaptosome fraction of the brains of DLB and MSA cases compared with those in the controls (Fig. 8b, h). Subsequently, we employed another antibody against aggregated α-Syn (5G4) to ascertain whether the α-Syn protein was increased in the synaptosome fraction. As the bands of SYT13 observed in the soluble fractions of DLB and MSA samples (Fig. 6a, b, white asterisk) largely corresponded to phosphorylated α-Syn, with a molecular weight of 100 kDa, we focused on 5G4-positive α-Syn with the same molecular weight. Consistent with the findings shown in Fig. 6c–e, the levels of aggregated form of α-Syn were markedly elevated in the synaptosomes of the brains of DLB (Fig. 8a arrowhead, c) and MSA cases (Fig. 8g arrowhead, i), compared with those in the samples from the control groups. Notably, the protein levels of 5G4-positive aggregated α-Syn, but not α-Syn monomers, were significantly correlated with the SYT13 protein levels (Fig. 8d, e, j, k). To examine alterations of extracellular vesicle release in synucleinopathies, extracellular vesicles were isolated from the temporal lobe of the same cases (DLB *n* = 5 and control *n* = 4; MSA *n* = 3 and control *n* = 3). Immunoblotting analysis using several extracellular vesicle-specific antibodies including those against CD9, CD63 and CD81, transmission electron microscopy, and interferometric light microscopy confirmed the isolation of extracellular vesicles (Fig. S1b, e–g). A significant decrease in the number of extracellular vesicles was observed in the temporal lobe homogenates from patients with DLB and MSA compared with those in the samples from the controls (Fig. 8f, l). These findings were also corroborated by nanoparticle tracking analysis (Video drop) (Fig. S6a–g).

### mRNA expression of SYT13 is dysregulated in synucleinopathies

**Fig. 9:**
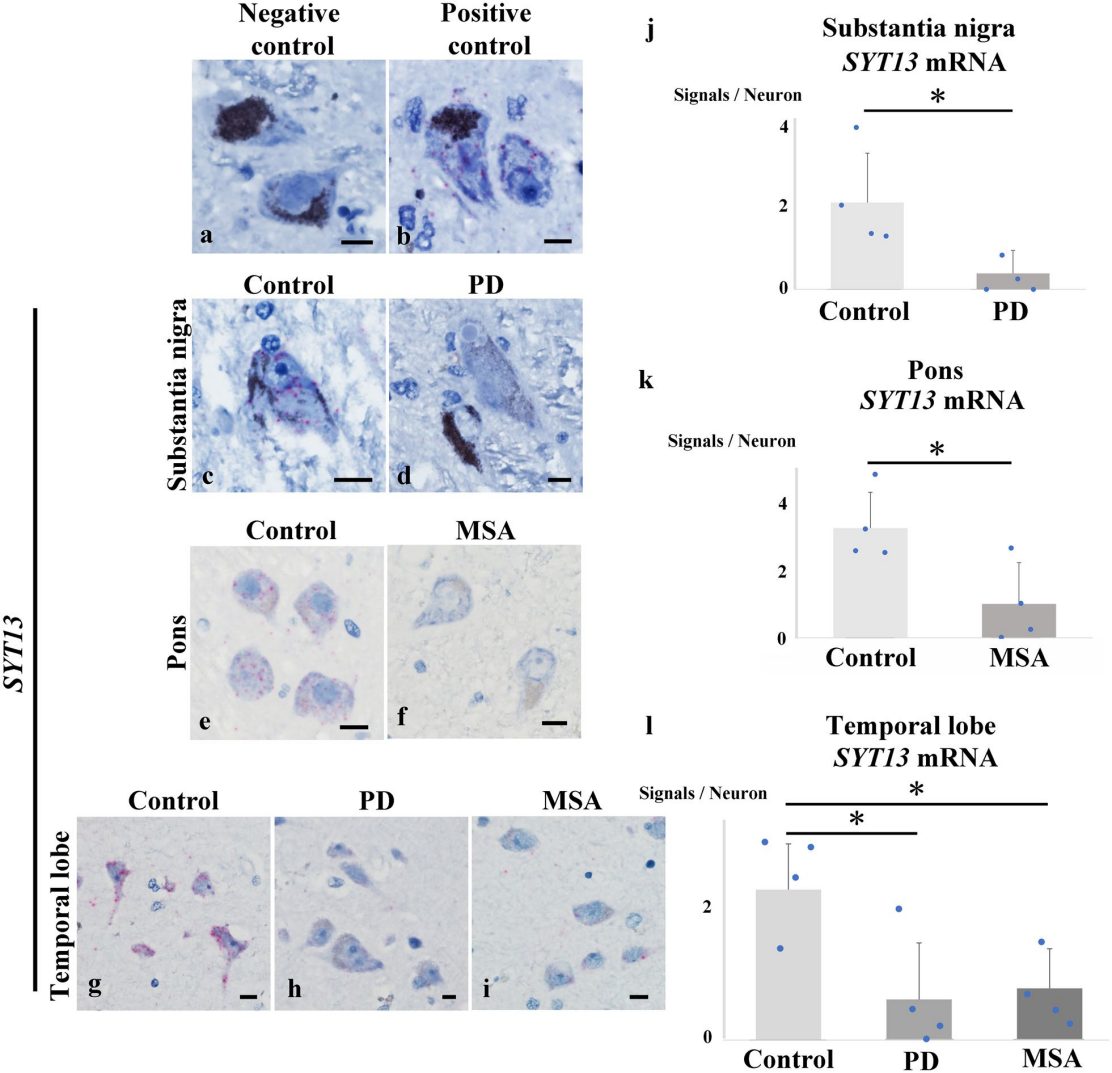
Alterations of *SYT13* mRNA expression in multiple brain regions of advanced-stage patients with synucleinopathies. RNAscope^®^ was performed to visualise *SYT13* mRNA. **a** No signals were observed when a negative control probe was employed. **b** A positive control probe showed signals of mRNA encoding a housekeeping protein Cyclophilin B. **c**–**i** mRNA expression levels of *SYT13* in advanced stages of synucleinopathies, comprising PD (*n* = 4) and control (*n* = 4) cases as well as MSA (*n* = 4) and control (*n* = 4) cases. **j** *SYT13* mRNA expression was reduced in the substantia nigra of PD cases compared to control cases. **k** *SYT13* mRNA expression was reduced in the pons of MSA cases compared to control cases. **l** Lower mRNA expression of *SYT13* in the temporal lobe of PD and MSA cases compared to controls. Mean ± SD, two-sample *t*-test (**j, k**) and one-way analysis of variance followed by the Tukey test (**l**). Scale bars, 10 μm. *P < 0.05

Finally, we investigated *SYT13* mRNA expression levels in the brains of individuals with synucleinopathies (Fig. 9a–l). This involved an analysis of PD (*n* = 4) and control (*n* = 4) cases, as well as MSA (*n* = 4) and control (*n* = 4) cases. All PD cases had α-Syn pathology in the brainstem, limbic area, and temporal lobe. We confirmed the presence of mRNA with a positive control probe for *Cyclophilin B* mRNA, but not with a negative control probe (Fig. 9a, b). We then studied the localisation of *SYT13* mRNA in the human brain. *SYT13* mRNA was found in neurons, but not in glial cells (Fig. S7a, b). In contrast to the increased *SYT13* mRNA expression observed following human α-Syn induction in the MSA mouse model (Fig. 1k), the *SYT13* mRNA expression was significantly reduced in multiple brain regions of PD and MSA patients, compared with the controls (Fig. 9j. k, l).

## Discussion

The present study provides new insights into the dysregulation of extracellular vesicle release associated with SYT13 in synucleinopathies. Our main findings include: 1) SYT13 at the synapses may regulate extracellular vesicle release in association with SYT1 and SNARE complexes; 2) pathological α-Syn, especially toxic β-sheet-rich oligomers, exhibit a higher degree of interaction with SYT13 than endogenous α-Syn; 3) SYT13 is mis-localised, together with abnormal α-Syn, from synapses to inclusions (Lewy bodies and GCIs); and 4) although both elevated and diminished protein levels of SYT13 lead to dysregulation of extracellular vesicle release in vitro, an increase in SYT13 level at the synapses may have a more deleterious effect on extracellular vesicle release in the brains of individuals with synucleinopathies. Previous in vitro research indicated that abnormal α-Syn might disrupt SNARE complex formation or SNARE-mediated vesicle fusion [16, 26–28]. In addition, some results appear contradictory, as many of these studies have been performed in vitro under non-physiological conditions [26–28]. To the best of our knowledge, our study was the first to demonstrate that aberrant interactions between abnormal α-Syn and SYT13 may lead to dysregulation of extracellular vesicle release in Lewy body diseases and MSA, as confirmed in post-mortem brain tissues. In our previous report, increased α-Syn oligomers in the hippocampus suppress long-term potentiation, one of the major mechanisms of memory storage, and decrease the number of dendritic spines in the MSA model [8]. Consistently, MSA cases with memory impairment exhibit a greater degree of accumulation of α-Syn oligomers in the medial temporal lobe, compared with that observed in cases without such impairment [8]. The findings of the present study further suggested that the synaptic dysfunction resulting from aberrant interactions between toxic β-sheet-rich α-Syn oligomers and SYT13 may explain some of the underlying causes of cognitive impairment associated with synucleinopathies. SYT13 may be a molecule that exhibits optimal functionality when present in the appropriate location and in the optimal quantity.

To understand the pathogenesis of synucleinopathies, two competing theories have been discussed for decades, namely, the toxic proteinopathy hypothesis and the proteinopenia hypothesis [51]. The former hypothesis posits that aggregated α-Syn becomes toxic and causes neurodegeneration. In contrast, the latter hypothesis proposes that α-Syn aggregates act as α-Syn sinks that decrease the concentration of physiologically active α-Syn at the synapses. In the present study, under physiological conditions, endogenous α-Syn and SYT13 may regulate extracellular vesicle release. However, the presence of abnormal α-Syn was associated with increased SYT13 level, compared to that with endogenous α-Syn. This interaction between abnormal α-Syn and SYT13 resulted in impaired vesicle release in synucleinopathies. Our data presented here support the “toxic gain-of-function of α-Syn” theory. However, according to previous reports, physiologically, endogenous α-Syn is a fundamental component of the SNARE complex assembly [16]. Therefore, it is also postulated that the physiological functions of α-Syn might also be lost when it is converted to abnormal forms. The toxic proteinopathy hypothesis versus the proteinopenia hypothesis may no longer be the sole explanation. A complex mixture of both toxic α-Syn gain-of-function and a loss of physiological functions may underlie the pathology.

Extracellular vesicles play a role in crucial processes within the nervous system [52], including neuronal survival, metabolism, synaptic plasticity, and stress responses. Recently, Chamberlain et al*.* [53] reported that extracellular vesicles support axonal energy metabolism via transfer of the NAD-dependent deacetylase SIRT2 from oligodendrocytes to axons. This transcellular delivery of SIRT2 maintains ATP homeostasis in axons through the deacetylation of mitochondrial adenine nucleotide translocases-1/2 [53]. Furthermore, Frühbeis et al. [54] observed that mice lacking proteolipid protein and 2′,3′-cyclic-nucleotide-3′-phosphodiesterase develop axonal degeneration, accompanied by reduced SIRT2 levels in extracellular vesicles, compared to wild-type mice. Thus, extracellular vesicles may be responsible not only for inter-neuronal communication but also for glial–neuronal crosstalk. In the present study, *SYT13* mRNA expression was identified predominantly in neurons (Fig. S7). Therefore, it is speculated that aberrant interactions between α-Syn and SYT13 may primarily affect the release of neuron-derived extracellular vesicles and inter–neuronal communication. However, further studies are warranted to elucidate how the dysregulation of neuron-derived extracellular vesicles affects the environment of glial cells in synucleinopathies.

To date, there are no disease-modifying therapies available for synucleinopathies in clinical settings. Our recent study demonstrated that intranasal administration of trehalose ameliorates memory impairment in the MSA mouse model by accelerating the transformation of α-Syn oligomers to fibrillar aggregates [9]. In line with previous reports supporting the neuroprotective role of inclusion body formation [11], α-Syn oligomers exert neurotoxic effects, whereas the formation of fibrillar aggregates in our previous study may be neuroprotective [9]. Recently, a monoclonal antibody against aggregated α-Syn, prasinezumab, designed to clear extracellular abnormal α-Syn, was reported to successfully enter the central nervous system and delay motor sign progression (Movement Disorder Society Unified Ranking Scale Part III) in patients with PD with faster motor progression [55]. These results support therapeutic approaches based on the toxic proteinopathy hypothesis. The use of antibodies that target α-Syn oligomers could be a promising therapeutic approach for maintaining SYT13 protein levels and the concentration of extracellular vesicles within the physiological range. The accumulation of abnormal α-Syn species in the medial temporal lobe is associated with cognitive impairment, but not with cell death [7, 8]. However, in regions susceptible to abnormal α-Syn, such as the striatonigral systems, dysregulated synaptic vesicle release may also be an initial step of a cascade of the pathological processes culminating in cell death. Therefore, this therapeutic approach is postulated to be beneficial for cognitive impairment as well as early symptoms in synucleinopathies.

In the present study, we observed that the mRNA expression patterns of *SYT13* exhibited contrasting profiles between the early and advanced stages of disease. In the MSA mouse model designed to replicate the early stages of MSA [8, 29], *SYT13* mRNA expression was increased in conjunction with human α-Syn induction. Conversely, in the advanced stages of human synucleinopathies, we observed a significant reduction in *SYT13* mRNA expression. This discrepancy could be attributed to the different characteristics of the mouse and human brains. However, disease duration may influence the transcriptional profiles of dopaminergic neurons in the midbrain of early- versus late-PD patients [56]. In fact, Kon et al*.* reported that *SNCA* transcripts are significantly reduced in dopaminergic neurons containing Lewy bodies in the substantia nigra of patients with PD [57]. The authors speculated that *SNCA* mRNA production may be exhausted in the terminal stage of the disease process. Together with the potential importance of tightly regulated SYT13 expression in maintaining extracellular vesicle release, it is plausible that *SYT13* mRNA expression may be downregulated or exhausted, as SYT13 protein levels were elevated in the brains of individuals with advanced stages of synucleinopathies. Conversely, PLA analysis revealed signals indicative of interactions between SYT13 and abnormal α-Syn. These signals were not limited to Lewy bodies and GCIs, but were diffusely distributed, with the majority being located in the neuropil. Furthermore, immunohistochemical analysis using antibodies against physiological and abnormal α-Syn (5G4) demonstrated a punctate pattern in the neuropil, in addition to the accumulation of α-Syn in Lewy bodies [58, 59]. The formation of Lewy bodies may result from conformational protein changes due to the anterograde and/or retrograde intraneuronal spreads of abnormal α-Syn [60]. Considering the elevated protein levels of SYT13 and its interactions with abnormal α-Syn at the synapses in the brains of DLB and MSA cases as illustrated in Fig. 8, PLA signals in the neuropil may indicate SYT13–α-Syn interactions at the synapses. The PLA signals incorporated into Lewy bodies and GCIs may represent the interactions between SYT13 and α-Syn molecules, which were sequestered from the synapse during the process of inclusion formation.

This study had some limitations. First, we performed in vitro analyses using SH-SY5Y cells. However, the cells did not form synapses under the current culture conditions, and therefore did not replicate the synapses of the human brain. Second, the methodology for isolating extracellular vesicles from human brain homogenates has yet to be established. However, in the present study the presence of extracellular vesicles was corroborated by immunoblotting with three different antibodies (CD9, CD81, and CD63), transmission electron microscopy, and interferometric light microscopy. In addition, membrane potential analysis further confirmed the changes of exocytosis. To isolate extracellular vesicles from culture supernatant and brain homogenates, magnetic beads conjugated with Tim4 were employed, as this bead specifically binds to phosphatidylserine on the surface of extracellular vesicles (MagCapture™). Further methodological development is required to improve the isolation of extracellular vesicles from brain homogenates.

In conclusion, we provide evidence that SYT13 regulates extracellular vesicle release. We also elucidated that toxic α-Syn oligomers might dysregulate extracellular vesicle release through binding to SYT13 in the brains of individuals with synucleinopathies. Maintaining SYT13 protein levels in cells by targeting α-Syn oligomers could thus be a promising therapeutic avenue, with particular potential for the treatment of the cognitive impairment associated with synucleinopathies.

## Supplementary Information

Additional file 1. Supplementary methods.

Additional file 2. **Fig. S1**. Identification of extracellular vesicles in the culture supernatant of SH-SY5Y cells and human brain homogenates. **Fig. S2** Identification of anti-synaptotagmin 13 (SYT13) antibodies suitable for immunoprecipitation. **Fig. S3** Protein–protein interactions of α-synuclein (α-Syn) and synaptotagmin 13 (SYT13) predicted using AlphaFold 3. **Fig. S4** Significant reduction in membrane capacitance in SH-SY5Y cells transfected with synaptotagmin 13 (SYT13) siRNA or overexpressed with SYT13 gene. **Fig. S5** Confirmation of interactions between synaptotagmin 13 (SYT13) and SYT1 or mammalian uncoordinated 18-1 (Munc18-1). **Fig. S6** Nanoparticle tracking analysis (Video drop) to assess extracellular vesicle release in patients with synucleinopathies.  **Fig. S7** mRNA encoding Synaptotagmin 13 (SYT13) is expressed in neurons of the human brain.

Additional file 3. **Table S1**. Demographic data of human cases

Additional file 4. **Table S2**. Information about antibodies.

Additional file 5. **Table S3**. The function and localisation of 10 vesicular transport proteins identified by transcriptome analysis in a mouse model of multiple system atrophy.

Additional file 6.** Table S4**. Top 10 upregulated or downregulated transcripts in a mouse model of multiple system atrophy.

Additional file 7. Predicted interaction between wild type α-Syn and SYT13. Predicted interaction between S129E α-Syn and SYT13

Additional file 8. Uncropped films.

## Data Availability

The raw data supporting the findings of the present study are available on request from the corresponding author. The raw data of the microarray have been deposited to GEO (http://www.ncbi.nlm.nih.gov/geo) under accession number GSE129531.

## Abbreviations

PD: Parkinson’s disease
DLB: Dementia with Lewy bodies
MSA: Multiple system atrophy
SNARE: Soluble N-ethylmaleimide-sensitive attachment protein receptor
GCI: Glial cytoplasmic inclusion
NCI: Neuronal cytoplasmic inclusion
SYT: Synaptotagmin
IP: Immunoprecipitation
Munc18-1: Mammalian uncoordinated 18-1
PLA: Proximity ligation assay
SNAP25: Synaptosomal-associated protein 25 kDa
RIMS3: Regulating synaptic membrane exocytosis 3
VTI1A: Vesicle transport through interaction with T-SNAREs 1A
