# Supplementary material for: Abnormal α-synuclein binds to synaptotagmin 13, impairing extracellular vesicle release in synucleinopathies
Source: Transl Neurodegener. 2025 Jun 23;14:32. doi: 10.1186/s40035-025-00493-6 (PMC12183919; doi:10.1186/s40035-025-00493-6)
Supplement: Supplementary file 1 — Additional file 1. Supplementary methods. [file 40035_2025_493_MOESM1_ESM.docx]

**Supplementary data**

***Histological analyses***

Immunohistochemistry was performed using 4-μm-thick, formalin-fixed, paraffin-embedded sections from the multiple brain regions of the MSA mouse model and human cases. In each analysis, the age, postmortem delay and fixation time were matched between the groups (Supplementary table 1). The cases for immunohistochemistry include Parkinson’s disease (PD): N = 7; dementia with Lewy bodies (DLB): N = 7; multiple system atrophy (MSA): N = 7; control: N = 7. We used antibodies against SYT13 (HPA046224; Sigma-Aldrich, MA, USA; 1:100 and NBP2-93419, Novus Biologicals, CO, USA; 1:100), Synaptosomal-associated protein 25 (SNAP25) (SP12, MA5-179609; Thermo Fisher Scientific, MA, USA; 1:100), Piccolo (HPA015858; Sigma-Aldrich; 1:250), Ras-related protein Rab7 (Rab7) (HPA006964; Sigma-Aldrich; 1:50), Rab11b (PA5-31348; ThermoFisher Scientific; 1:100), Rab23 (NBP1-86367; Novus Biologicals; 1:400), Regulating synaptic membrane exocytosis 3 (RIMS3) (GTX31334; GeneTex, CA, USA; 1:400), RIMS4 (C18307; Assay Bioteck, CA, USA; 1:100), Exophilin5 (22199-1-AP; Proteintech, IL, USA; 1:100) and human α-synuclein (syn211; ab80627, Abcam, Cambridge, UK; 1:1,000). The sections were subjected to immunohistochemical staining using the avidin-biotin-peroxidase complex method. Diamino benzidine was used as the chromogen and haematoxylin was used for counterstain. To evaluate the SYT13 positive rate in α-synuclein-positive Lewy bodies or glial cytoplasmic inclusions, four-μm serial sections were prepared. Immunohistochemical staining was performed using serial sections with anti-SYT13 (HPA046224 and NBP2-93419) and anti-α-synuclein (syn211) antibodies. The positive ratio was calculated as the number of SYT13-positive Lewy bodies or glial cytoplasmic inclusions divided by the number of α-synuclein-positive Lewy bodies or glial cytoplasmic inclusions. The evaluation was conducted by two experienced neuropathologists (Y.M. and K.W.). For double-label immunofluorescence staining using human cases of PD and MSA, paraffin sections were incubated overnight at 4°C with a mixture of mouse anti-human α-synuclein (syn211; 1:500) and rabbit anti-SYT13 (HPA046224; 1:50 and NBP2-93419; 1:50) antibodies. Subsequently, the sections were rinsed and incubated with either anti-mouse IgG tagged with Alexa Fluor 488 (A21202; Invitrogen, CA, USA; 1:200), or anti-rabbit IgG tagged with Alexa Fluor 555 (A31572, Invitrogen, 1:200), for one hour at 37°C. The sections were then mounted with Vectashield (Vector Laboratories Inc., Burlingame, CA, USA) and examined with a confocal fluorescence microscope (EZ-Ci, Nikon, Tokyo, Japan). Demographic data of human cases are elucidated in supplementary table 1. The primary and secondary antibodies utilized in the present study are thoroughly documented in supplementary table 2.

***Base scope and RNAScope assays***

To visualise mRNA for human α-synuclein in oligodendrocytes, we performed Base scope assay using a customised 2ZZ probe designed to recognise mRNA for human α-synuclein (310-402 bp of NM_001146055.2) (323970; 1173011-C1; 320269; Advanced Cell Diagnostics,CA, USA). Four-μm-thick, formalin-fixed, paraffin-embedded sections from the brain of mouse (N = 1, 43 weeks old, male) were used for this analysis. Following deparaffinisation, the sections were treated with RNAscope® Hydrogen Peroxide for a period of 10 minutes at room temperature. This was followed by a further 15 minutes of treatment with RNAscope® Target Retrieval Reagents at a temperature of 98-102 ℃, and then a period of 30 minutes with RNAscope® Protease IV at a temperature of 40 ℃. Subsequently, the negative control probe or probe for human α-synuclein mRNA was hybridised for a period of two hours at 40 degrees Celsius. Subsequently, BaseScope™ v2 AMP1 was hybridised for a period of 30 minutes at 40 degrees Celsius, followed by AMP2 for a further 30 minutes, AMP3 for 15 minutes, AMP4 for 30 minutes, AMP5 for 30 minutes, AMP6 for 15 minutes, AMP7 for 30 minutes at room temperature, and finally AMP8 for 15 minutes at room temperature. Following each hybridization process, the sections were washed with 1x RNAscope™ Wash Buffer. Subsequently, the sections were incubated for 10 minutes at room temperature with BaseScope™ Fast RED-A and -B. Subsequently, the sections were counterstained with a 50% solution of Haematoxylin, followed by 0.02% ammonia water. Subsequently, the sections were mounted with VectaMount (Vector Laboratories Inc.). The manufacturer’s user manual is available (<https://acdbio.com/sites/default/files/USM-323900%20BaseScope%20Detection%20Reagent%20v2%20_06082018.pdf>).

To visualise mRNA of *SYT13* in the brain, we performed RNAScope assay using RNAScope^TM^ positive and negative control probes and a probe that recognises mRNA for *human SYT13* (313901; 310043; 552271; Advanced Cell Diagnostics, CA, USA). For RNAScope assay, we used four-μm-thick, formalin-fixed, paraffin-embedded sections from several brain regions (temporal lobe, midbrain and pons) of patients with PD (N = 4) and MSA (N = 4), and controls (N = 4). The age, postmortem delay and fixation time were all matched between the groups. Following deparaffinisation, the sections were treated with RNAscope® Hydrogen Peroxide for 10 minutes at room temperature, followed by RNAscope® Target Retrieval Reagents for 15 minutes at 98-102 ℃ and RNAscope® Protease IV for 30 minutes at 40 ℃. Subsequently, the negative control probe or probe for human *SYT13* mRNA was hybridised for two hours at 40 ℃. RNAScope™ v2.5 AMP1 was then hybridised for a period of 30 minutes at 40 ℃, AMP2 for 15 minutes at 40 ℃, AMP3 for 30 minutes at 40 ℃, AMP4 for 15 minutes at 40 ℃, AMP5 for 30 minutes at room temperature and AMP6 for 15 minutes at room temperature. Following each hybridization process, the sections were washed with 1x RNAscope™ Wash Buffer. Signals were detected with 1:60 ratio of Fast RED-B to Fast RED-A. The manufacturer’s user manual is available (https://www.cosmobio.co.jp/product/uploads/document/ADC_rnascope-singleplex-assay_manual_2.pdf).

***Cell culture***

HEK293 and SH-SY5Y cells were obtained from the Japanese Collection of Research Bioresources Cell Bank (Osaka, Japan) and American Type Culture Collection (Manassas, VA, USA), respectively. HEK293 cells were maintained in Dulbecco's modified Eagle medium (DMEM) supplemented with 10% foetal bovine serum (FBS) and antibiotics. SH-SY5Y were generated that stably express human α-synuclein, as reported previously [1]. As FBS contains extracellular vesicles, a medium was supplemented with extracellular vesicle-free FBS (EXO-FBSHI-50A-1, System Biosciences, Palo Alto, CA, USA) for SH-SY5Y cell culture.

***Isolation of extracellular vesicles***

SH-SY5Y cells were washed twice with 1x phosphate-buffered saline and subsequently cultured in a medium supplemented with extracellular vesicle-free FBS (EXO-FBSHI-50A-1, System Biosciences, Palo Alto, CA, USA), and transfected with Silencer® Select *siRNA* mammalian uncoordinated 18-1 (concentration: 25pmol/9.5cm^2^) (AS02MN4G; Thermo Fisher Scientific) Silencer® Select *siRNA SYT1* (concentration: 25pmol/9.5cm^2^) (AS02MU6X; Thermo Fisher Scientific), Silencer® siRNA *SYT13* (concentration: 25pmol/9.5cm^2^) (AM16708, Thermo Fisher Scientific), *siRNA control* (concentration: 25pmol/9.5cm^2^) (D-001810-10-20, Dharmacon, CO, USA) or 0.1 μg of *FLAG-tagged full-length SYT13 cDNA*. ScreenFect™ siRNA (299-75001; FUJIFILM Wako; Osaka Japan) was used for the transfection of siRNA. 48 after transfection, extracellular vesicles were isolated from the cell culture supernatant of SH-SY5Y cells treated with *FLAG-tagged full-legth SYT13 cDNA*, *siRNA SYT1, siRNA SYT13* or *siRNA control*. MagCapture™ Exosome Isolation Kit (290-84103; FUJIFILM Wako) was used for the isolation in accordance with the manufacturer’s protocol (<https://labchem-wako.fujifilm.com/jp/product_data/docs/04095588_doc01.pdf>). The samples were then lysed with sample buffer (75 mM Tris-HCl, pH 6.8, 4% sodium dodecyl sulfate, 25% glycerol, 5% β-mercaptoethanol).

Extracellular vesicles were also isolated from the brain homogenates of DLB (N = 4) and control cases (N = 5), and MSA (N = 3) and control cases (N = 3). Brain samples were gently homogenised with 0.01M phosphate buffer saline, followed by isolation of extracellular vesicles with MagCapture^®^, as described above.

The presence of extracellular vesicles obtained from the culture supernatant and human brain homogenate was examined by immunoblotting using antibodies against CD9 (4H7B9, 60232-1-IG; Proteintech, IL, USA; 1:1,000), CD63 (25682-1-AP; Proteintech; 1:1,000) and CD81 (B-11, sc-166029; Santa Cruz Biotechnology, MA, USA; 1:250), transmission electron microscopy (JEOL Ltd.; Tokyo, Japan), and interferometric light microscopy (Video drop; MEIWAFOSIS CO., LTD.; Tokyo, Japan) (Supplementary Fig. 1a-g). To conduct interferometric light microscopy, samples of a volume of 5 µL were applied to the Videodrop, which automatically calculated the concentration of extracellular vesicles. The methodologies regarding immunoblotting and transmission electron microscopy are elucidated in the following paragraphs.

***Immunoblotting***

As reported previously [2,3], Immunoblotting was performed on extracellular vesicles, cell lysates and the temporal lobe homogenates of human cases that had been dissected out at autopsy and frozen rapidly at −70°C. In each analysis (immunoblotting, fraction analyses, filter trap assay and isolation of extracellular vesicles) using human cases, age, ratio of genders and postmortem delay were matched between the groups (Supplementary table 1). All antibodies used for these analyses were also shown in supplementary table 2. Cases of DLB (N = 6) and control (N = 6), and cases of MSA (N = 6) and control (N = 6) were examined for immunoblotting. The primary and secondary antibodies utilized in the present study are thoroughly documented in the supplementary table 2.

***Transmission electron microscope***

The presence of extracellular vesicles in the culture supernatant of SH-SY5Y cells and human temporal lobe homogenate (obtained from a 72-year-old male) was examined using a transmission electron microscope. Extracellular vesicles from human breast milk were employed as a positive control (EXHM100L; Cosmo Bio Co., LTD.; Tokyo, Japan). The samples containing extracellular vesicles were applied to film-coated and carbon-coated copper grids (Nissin EM; Tokyo, Japan) and incubated for a period of five minutes. Any excess samples were then removed from the edge of the grid using filter paper. Subsequently, the grids were washed on three occasions with filtered distilled water and blotted onto a filter paper. Negative staining was conducted with 2% filtered uranyl acetate for a period of five minutes. Any excess dye was removed from the surface of the grid by blotting onto a filter paper, followed by three washes with filtered distilled water. The grid was then air-dried for a period of ten minutes. Photomicrographs were obtained using a JEOL 1230 electron microscope (JEOL Ltd.; Tokyo, Japan) at an accelerating voltage of 80 kV.

***Transcriptome analysis***

RNA was isolated from the right cerebral hemisphere of the MSA mouse model (N = 3, 12 weeks old, male) and proteolipid protein-Cre recombinase/oestrogen receptor or α-synuclein-flox mice as a control (N = 3, 12 weeks old, male). We then performed transcriptome-wide analysis by microarray (Clariom D assay, mouse, Thermo Fisher Scientific, MA, USA) as reported previously [4].

***Proximity ligation assay***

As reported previously [2,3], proximity ligation assay (PLA) (DUO92012; Merck Millipore, MA, USA) was performed using 4-μm-thick, formalin-fixed, paraffin-embedded sections to detect interaction between α-synuclein and SYT13. For this analysis, we used several brain regions (temporal lobe, midbrain and pons) of patients with PD (N = 4) and MSA (N = 4), and controls (N = 4). The age, postmortem delay and fixation time were all matched between the groups. The sections were also subjected to heat retrieval for 10 minutes in 10 mmol/L citrate buffer (pH 6.0). All sections were treated with blocking solution at 37°C for one hour, followed by incubation with primary a combination of rabbit anti-SYT13 (NBP2-93419; Novus biologicals; 1:50) and mouse anti-endogenous α-Syn antibodies (syn211, ab80627; Abcam; 1:100) or mouse anti-phosphorylated α-Syn (#64, 015-25191; FUJIFILM Wako; 1:500) at 37°C for one hour, and then at 4°C overnight. The sections were treated with ligation solution containing ligase at 37°C for one hour, after which they were further treated with amplification reagents, including polymerase, at the same temperature for a further 2 hours. The signals were incubated with substrate solutions at room temperature for 20 minutes, and visualised with detection solution at room temperature for 1 hour. Haematoxylin was employed as a counterstain. The manufacturer’s protocol is available (<https://www.sigmaaldrich.com/JP/en/technical-documents/protocol/protein-biology/protein-and-nucleic-acid-interactions/duolink-brightfield-user-manual>).

***Prediction of protein-protein interaction***

Protein-protein interaction was predicted using AlphaFold3 [5]. Wild-type α-synuclein (α-Syn) (P37840) or S129E mutants and synaptotagmin13 (SYT13) (Q7L8C5) were used as the subject and object, respectively. The model was selected from a number of candidates that satisfied the following criteria: the retention of the α-helix structure of α-Syn N-terminal and the C2A or C2B domain of SYT13. Structural indications (coloured by chain and domain, shown as a cartoon) and intermolecular distances (Å) were calculated using PyMOL (Version 2.6.0).

***Gene construct***

FLAG-tagged full-length *human SYT13* and deletion mutants of Flag-tagged *human SYT13* cDNA were amplified from *SYT13* (NM_020826) ORF Vector (OL01313882APP, Amerigo Scientific, NY, USA) using PrimeStar Max DNA Polymerase (R045A, TAKARA BIO INC., Shiga, Japan) and specific pairs of primers: Full-length *SYT13* (Fw: 5’-tgacgatgacaagcttatggtgctgtcggtgcct-3’; Rv: 5’-atctatcgatgaattcttacaggtgcagctggtg-3’), Δ1-159 *SYT13* (Fw: 5’-tgacgatgacaagcttatggtgctgtcggtgcct-3’; Rv: 5’-atctatcgatgaattcctaggcctggttccaact-3’), C2A *SYT13* (Fw: 5’-tgacgatgacaagcttcccaaactccactactgc-3’; Rv: 5’-atctatcgatgaattcctacaagctcgccccactg-3’) and C2B *SYT13* (Fw: 5’-tgacgatgacaagcttgctggagaggtcctacta-3’; Rv: 5’-atctatcgatgaattcttacaggtgcagctggtg-3’). The products were inserted into the p3xFLAG-CMV7.1 cut by HindIII and EcoRI according to the protocol provide by In-Fusion HD Cloning Kit (Z9648N, TAKARA BIO INC.). The inserted DNAs were analysed and confirmed by polymerase chain reaction using the following primer pair (Fw: caaggatgacgatgacaagc; Rv: ttttattaggacaaggctggtg) and sequencing.

***Immunoprecipitation***

For immunoprecipitation using HEK293 cells, FLAG-tagged full-length *human SYT13*, deletion mutants of FLAG-tagged *human SYT13* (Δ1-159, C2A and C2B) and *human α-Syn* cDNAs were prepared as described above. Serine residue at position 129 was substituted with glutamic acid (E) to create a phosphorylation-mimic mutant (S129E α-Syn) [2]. HEK293 cells were transfected with a cDNA containing 0.25 μg of S129E *α-Syn* and a combination of 0.1 μg of Flag-tagged *SYT13* (full-length, Δ1-159, C2A or C2B), incubated for 24 hours. X-tremeGENE 9® (6365779001; Roche, Basel, Switzerland) was used for this transfection. The pre-immunoprecipitated lysate was diluted twofold with immunoprecipitation (IP) buffer and used as an input sample. The lysate was then immunoprecipitated with anti-FLAG (20543-1-AP; Proteintech) or IgG alone coupled to the surface of Dynabeads™ M-270 Epoxy beads (14321D; Thermo Fisher Scientific) according to the manufacturer’s protocol (<https://www.thermofisher.com/jp/ja/home/references/protocols/proteins-expression-isolation-and-analysis/antibody-protocol/dynabeads-co-immunoprecipitation-kit.html>). The lysate was then analysed by immunoblotting.

For immunoprecipitation using human brains (DLB: N = 5 and control: N =5; MSA: N = 3 and control: N = 3; control for the identification of suitable antibodies for immunoprecipitation: N = 2), the pre-immunoprecipitated lysate from the temporal lobe of human cases was diluted twofold with IP buffer and used as an input sample. A human blood sample (N = 1) was also used as a negative control. The brain lysate was then immunoprecipitated with three different rabbit anti-SYT13 antibodies (NBP2-93419; Novus Biologicals, MBS9606007; MyBioSource, California, SD, USA, ABIN2840354; Antibodies-online.com, PA, USA) or rabbit IgG antibody (AP132; Sigma-Aldrich) coupled to the surface of Dynabeads™ M-270 Epoxy beads (14321D, ThermoFisher Scientific) according to the manufacturer’s protocol described above. The lysate was then analysed by immunoblotting. No bands were found in the input and IP sample of human blood immunoprecipitated with MBS9606007, and in the IP sample of human brain homogenate immunoprecipitated with rabbit anti IgG antibody (Supplementary Fig. 2a, b). However, SYT13 in the IP sample of the human brain was successfully immunoprecipitated with NBP2-93419 and MBS9606007, but not with ABIN2840354 (Supplementary Fig. 2b, arrowhead).

***Fraction analysis***

For fraction analysis using human brains (DLB: N = 3 and control: N = 3; MSA: N = 3 and control: N = 3), frozen tissues were weighed and sequentially extracted with buffers of increasing detergent strength using a modified protocol described previously [3]. In brief, brain samples were homogenised with 10 volumes of buffer A (30 mM Tris-HCl, pH 7.5, 10% glycerol, 150 mM NaCl) with an EDTA-free protease inhibitor cocktail (Roche Applied Science, IN, USA) and centrifuged at 1,000 x g for 10 minutes at 4℃ (TBS fraction). An additional equal volume of buffer A containing 0.2% Triton X-100 was then added to the TBS-insoluble pellet. Subsequently, the samples were incubated for 30 minutes at 37°C and centrifuged at 14,500 x g for 30 minutes at 4°C (supernatant: Triton fraction). The Triton-insoluble pellet was homogenised with an equal volume of buffer A containing 1% sarkosyl and then incubated for 30 minutes at 37°C. Subsequently, the homogenate was centrifuged at 14,500 x g for 30 minutes at 4℃ (supernatant: sarkosyl fraction). The sarkosyl-insoluble pellet was incubated CHAPS for 30 minutes at 37℃ and centrifuged at 14,500 x g for 30 minutes at 4℃ (supernatant: CHAPS fraction). Finally, the pellet was subjected to sonication and completely dissolved in 8M urea buffer, thereby forming the urea fraction. These fractionated brain lysates were analysed by immunoblotting using anti-SYT13 antibody (ab154695; Abcam; 1:1,000) and anti-phosphorylated α-Syn antibody (EP1536Y, ab51253, Abcam, 1:1,000). In addition, using the temporal lobes from cases of DLB (N = 1) and MSA (N = 1) and control (N = 1), cases of DLB (N = 5) and control (N = 4), and cases of MSA (N = 3) and control (N= 3), synaptosome was also isolated using Minute™ Synaptosome Isolation Kit (SY-052; Invent Biotechnologies, MN, USA) in accordance with the manufacturer’s protocol (https://cdn.shopify.com/s/files/1/0063/4575/2687/files/52-SY-052_v2.pdf?v=1639496134). The synaptosome was then assessed by immunoblotting.

***Filter trap assay***

Filter-trap analysis was performed using fractionated lysates (TBS, sarkosyl and urea fractions) of the temporal lobe of MSA cases (N = 6) and controls (N = 6), as reported previously [3]. The same volume of samples were applied to a 0.22-mm cellulose acetate membrane (C020A142C; Advantech, Taipei, Taiwan) on a slot blot apparatus (Bio-Rad, CA, USA) using a vacuum manifold. After the membrane had been washed with TBS containing 0.1% Tween-20, it was incubated with anti-aggregated α-Syn (26F1; Syngle therapeutics, https://www.syngletherapeutics.com; 1:2,000). Levels of protein expression were quantified using ImageJ.

***Semi-quantitative and quantitative analysis***

For quantitative analysis, the levels of protein expression were quantified using the ImageJ software （National Institutes of Health, Bethesda, MD, USA）. For semi-quantitative analyses, ten areas per case were randomly selected and photographed at x40 magnification. Subsequently, the PLA signals were quantified in pixels using the ImageJ Fiji software (National Institutes of Health). To assess mRNA levels of SYT13 in neurons, ten areas per case were randomly selected and photographed at x20 magnification. In RNAscope analysis, a cell with a round nucleus, containing a prominent nucleolus, and Nissl substance was defined as a neuron. Cells without these features were defined as glial cells. The number of positive signals in neurons was counted by two experienced neuropathologists (YM and KW).

***Membrane capacitance measurements***

The patch-clamp technique was performed to measure membrane capacitance. The SH-SY5Y cells were plated on Cell Disk LF (Sumitomo Bakelite, Tokyo, Japan). After the transfection of *SYT13* plasmid DNA (299-75001; FUJIFILM Wako) or *SYT13* siRNA (AM16708, Thermo Fisher Scientific), the Cell Disk was placed into the recording chamber and membrane capacitance was measured by whole-cell patch-clamp mode. The concentrations of *SYT13* plasmid DNA and *SYT13* siRNA were described above. 0.05μg of Green Fluorescent protein was also co-transfected to help identify transfected SH-SH5Y cells. The external recording solution contained (mM) 120 NaCl, 4 KCl, 1 CaCl_2_, 0.3 MgCl_2_, 0.4 MgSO_4_, 0.5 NaH_2_PO4, 0.5 Na_2_HPO_4_, 29 NaHCO_3_, and 17.5 glucose. The temperature of the external solution was controlled at 30-32 °C. Patch electrodes were fabricated from 1.5 mm diameter borosilicate capillary tubing (GD-1.5; Narishige, Tokyo, Japan) using a vertical puller (PP-830, Narishige). The electrode resistance ranged from 4 to 8 MΩ when the electrode was filled with a solution containing (in mM) 130 K-gluconate, 10 KCl, 10 HEPES, 0.4 EGTA, 2 MgCl_2_, 2 Mg-ATP, and 0.3 Na_2_-GTP. The pH was adjusted to 7.25 with KOH. Voltage clamp were performed using an amplifier (Multiclamp 700B, Molecular Devices, CA, USA) and digitizer (Digidata 1440A, Molucular devices). Pipette capacitance was compensated before going to whole-cell mode. After whole-cell mode was obtained, membrane voltage was clamped to –60 mV. Cell membrane capacitance was derived from acquisition software, Clampex 10.2, membrane-test function (Molecular Devices).

**Reference**

1. Nozaki S, Hijioka M, Wen X, Iwashita N, Namba J, Nomura Y, et al. Galantamine suppresses α-synuclein aggregation by inducing autophagy via the activation of α_7_ nicotinic acetylcholine receptors. J Pharmacol Sci. 2024; 156(2): 102-114.
2. Miki Y, Tanji K, Mori F, Tatara Y, Utsumi J, Sasaki H, et al. AMBRA1, a novel α-synuclein-binding protein, is implicated in the pathogenesis of multiple system atrophy. Brain Pathol. 2018; 28(1): 28-42.
3. Miki Y, Tanji K, Shinnai K, Tanaka MT, Altay F, Foti SC, et al. Pathological substrate of memory impairment in multiple system atrophy. Neuropathol Appl Neurobiol 2022; 48(7): e12844.
4. Furukawa T, Shimoyama S, Miki Y, Nikaido Y, Koga K, Nakamura K, et al. Chronic diazepam administration increases the expression of Lcn2 in the CNS. Pharmacol Res Perspect 2017; 5(1): e00283.
5. Abramson J, Adler J, Dunger J, Evans R, Green T, Pritzel A, et al. Accurate structure prediction of biomolecular interactions with AlphaFold 3. Nature. 2024; 630(8016): 493-500.
