# Supplementary material for: Abnormal α-synuclein binds to synaptotagmin 13, impairing extracellular vesicle release in synucleinopathies
Source: Transl Neurodegener. 2025 Jun 23;14:32. doi: 10.1186/s40035-025-00493-6 (PMC12183919; doi:10.1186/s40035-025-00493-6)
Supplement: Supplementary file 2 — Additional file 2. Fig. S1. Identification of extracellular vesicles in the culture supernatant of SH-SY5Y cells and human brain homogenates. Fig. S2 Identification of anti-synaptotagmin 13 (SYT13) antibodies suitable for immunoprecipitation. Fig. S3 Protein–protein interactions of α-synuclein (α-Syn) and synaptotagmin 13 (SYT13) predicted using AlphaFold 3. Fig. S4 Significant reduction in membrane capacitance in SH-SY5Y cells transfected with synaptotagmin 13 (SYT13) siRNA or overexpressed with SYT13 gene. Fig. S5 Confirmation of interactions between synaptotagmin 13 (SYT13) and SYT1 or mammalian uncoordinated 18-1 (Munc18-1). Fig. S6 Nanoparticle tracking analysis (Video drop) to assess extracellular vesicle release in patients with synucleinopathies. Fig. S7 mRNA encoding Synaptotagmin 13 (SYT13) is expressed in neurons of the human brain. [file 40035_2025_493_MOESM2_ESM.docx]

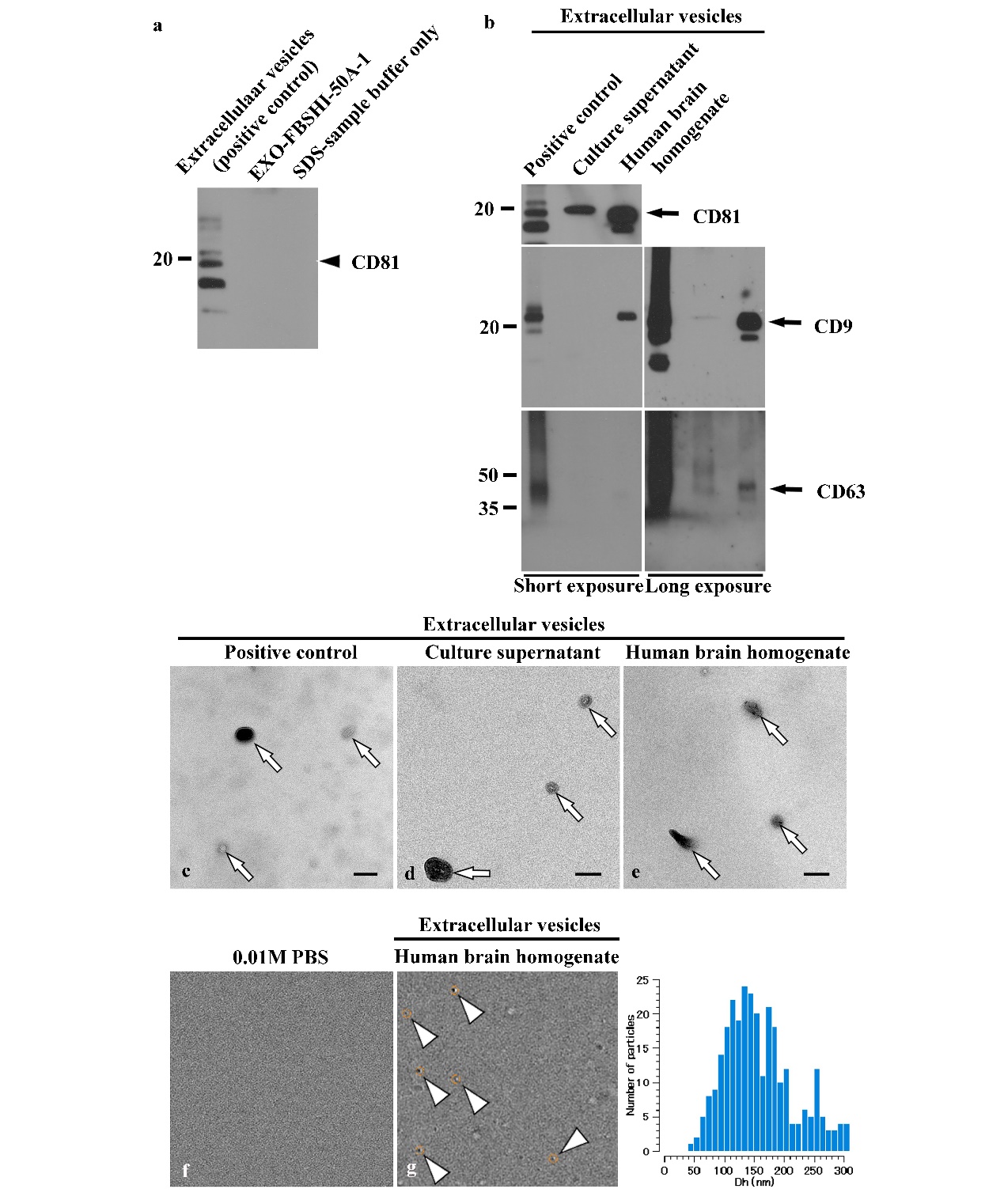


**Fig. S1 Identification of extracellular vesicles in the culture supernatant of SH-SY5Y cells and human brain homogenates.**

1. Immunoblotting showed no extracellular vesicles in extracellular vesicle-free foetal bovine serum (EXO-FBSHI-50A-1). (b) Immunoblotting using antibodies against extracellular vesicles (CD81, CD9, and CD63) confirmed the presence of extracellular vesicles obtained from the culture supernatant of SH-SY5Y cells and human brain homogenates. (c–e) Transmission electron microscopy further confirmed the presence of extracellular vesicles with a diameter of 50-150 nm in the culture supernatant and human brain homogenate. (f, g) Nanoparticle tracking analysis (Video drop) also showed the isolation of extracellular vesicles with a modal diameter of 135 nm in the brain homogenate of a control case (g), but no such vesicles in 0.01 MPBS (f). Bars = 100 nm (c-e).


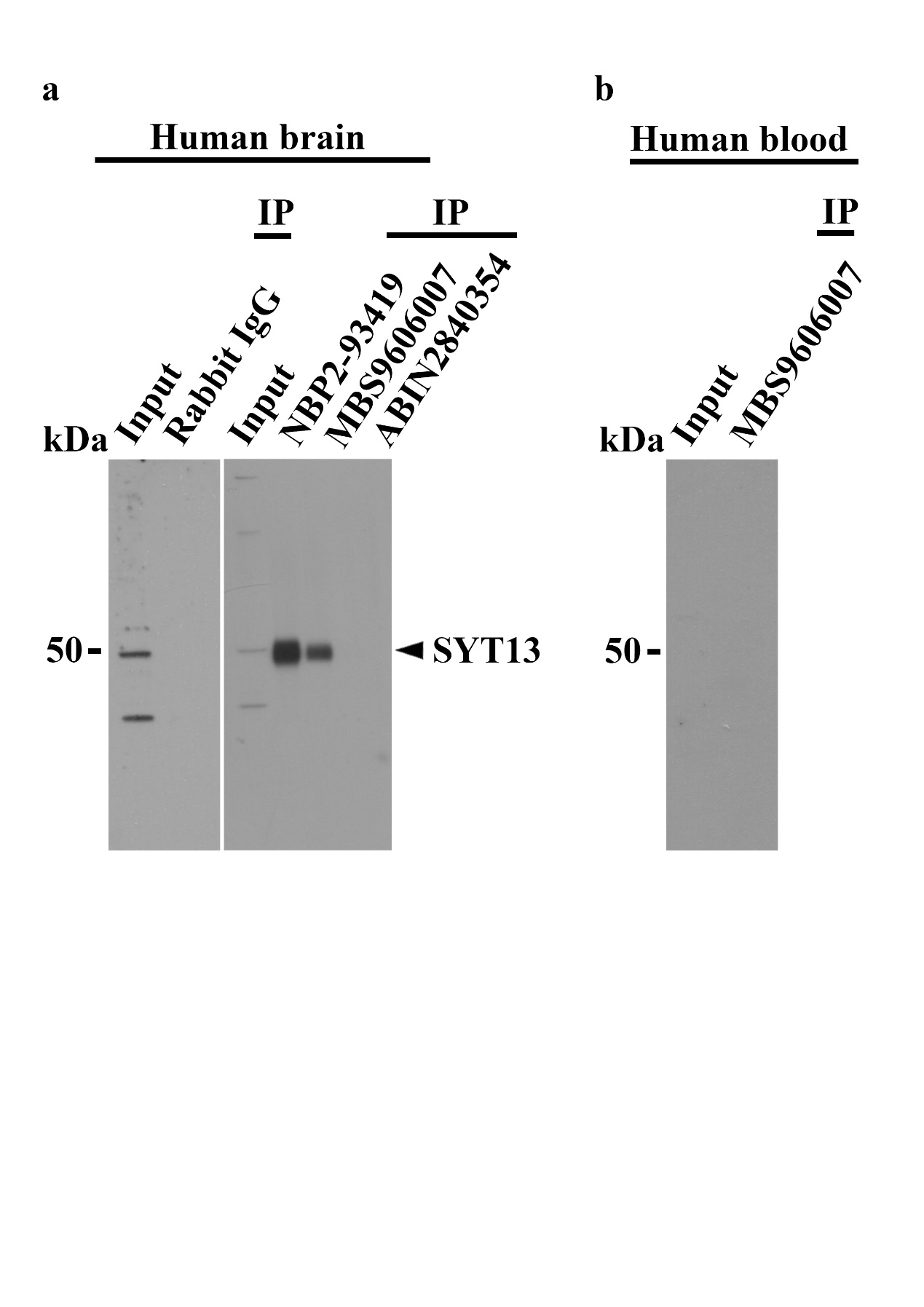


**Fig. S2 Identification of anti-synaptotagmin 13 (SYT13) antibodies suitable for immunoprecipitation.**

1. To identify which anti-SYT13 antibody is suitable for immunoprecipitation, the human brain temporal lobe homogenates were immunoprecipitated with three rabbit anti-SYT13 (NBP2-93419; MBS9606007; ABIN2840354) and anti-rabbit IgG antibodies. No bands were seen in the negative control (Rabbit IgG). However, a band of the molecular weight of 50 kDa for SYT13 was observed in the input. SYT13 was successfully immunoprecipitated with NBP2-93419 and MBS9606007 (arrowhead). In addition, SYT13 was not immunoprecipitated with ABIN2840354. (b) To further confirm the specificity of the band (a, arrowhead), a human blood sample was immunoprecipitated with MBS9606007. Immunoblotting analysis showed no bands at 50 kDa in the input and IP samples. Abbreviation: IP = immunoprecipitation.


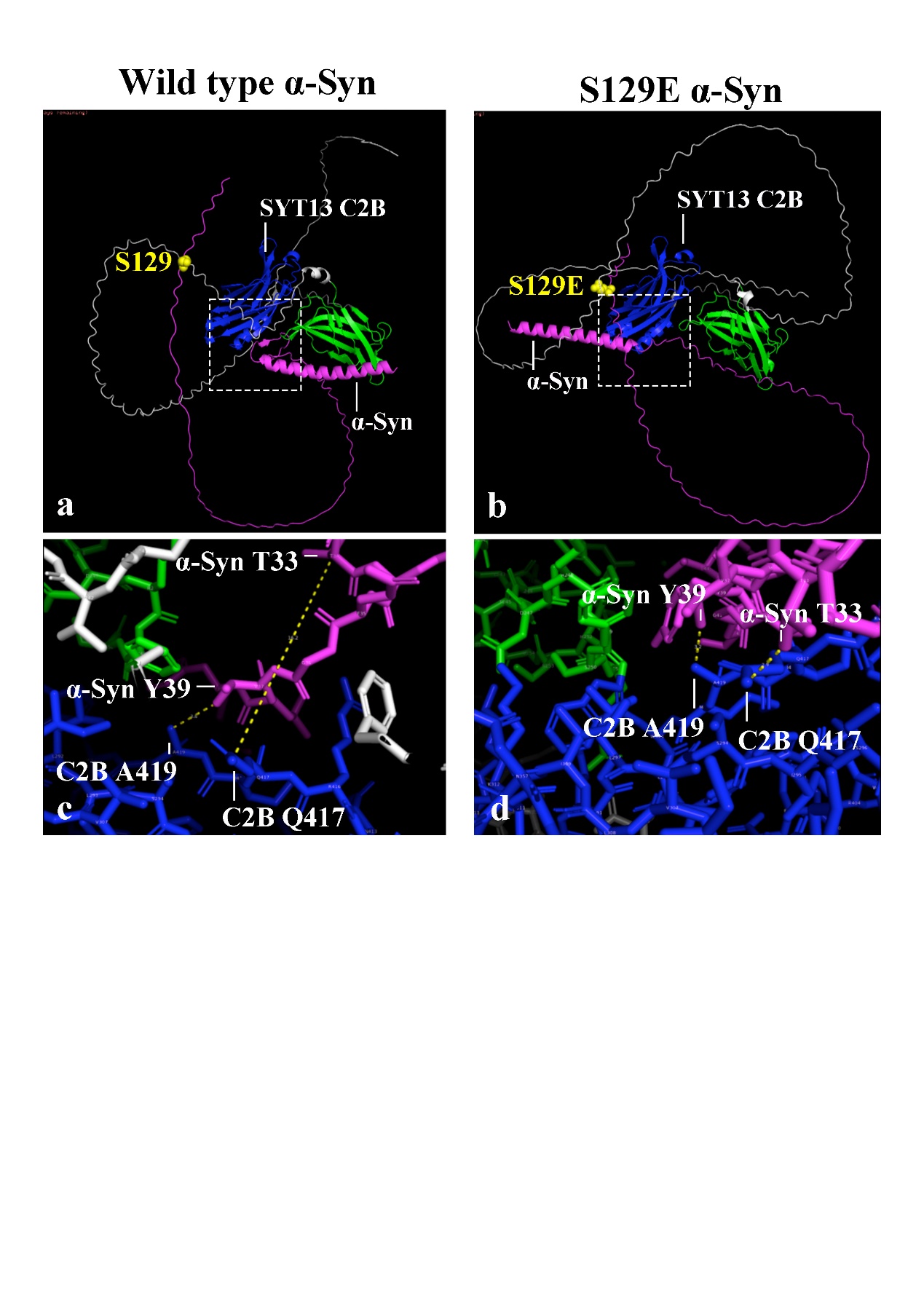


**Fig. S3 Protein–protein interactions of α-synuclein (α-Syn) and synaptotagmin 13 (SYT13) predicted using AlphaFold 3.**

(a, b) The structural composition of SYT13 and wild-type α-Syn (a) or S129E α-Syn

(b), a phosphorylation-mimic mutant in which serine residue (S) at position 129 was substituted with glutamic acid (E). (c) A magnified view of a white square (a) showed weak interaction between SYT13 and wild-type α-Syn with distances between wild-type α-Syn T33 and SYT13 C2B Q417, and α-Syn Y39 and SYT13 C2B Q419 being 16.2 Å and 3.6 Å, respectively. (d) A magnified view of a white square (b) demonstrated a binding interaction between S129E α-Syn and SYT13 C2B, with distances between S129E α-Syn T33 and SYT13 C2B Q417 and α-Syn Y39 and SYT13 C2B Q419 being 2.9 Å and 3.2 Å, respectively. Magenta: human wild-type α-Syn or human S129E α-Syn; white: the N-terminal region (1-157) of SYT13; green: the C2A domain (158-275); blue: the C2B domain (287-422); yellow: serin residue at 129 (S129 for wild type) or substitution of S with E for S129E mutants; grey: non-functional region.


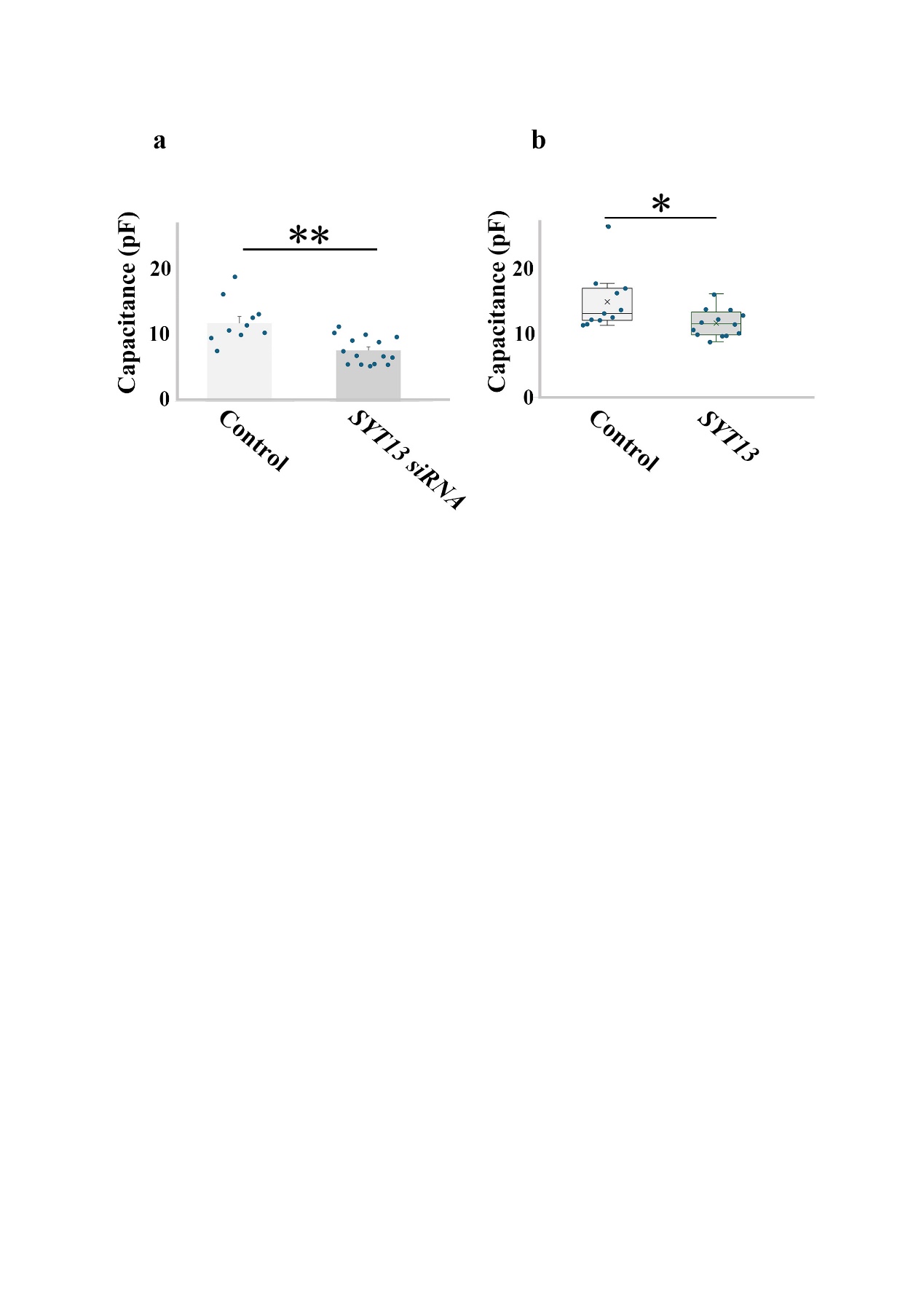


**Fig. S4 Significant reduction in membrane capacitance in SH-SY5Y cells transfected with *synaptotagmin 13 (SYT13)* siRNA or overexpressed with *SYT13* gene.**

To corroborate the alterations in exocytosis in SH-SY5Y cells treated with *SYT13* siRNA or overexpressed with *SYT13* gene, the membrane capacitance was examined. (a, b) A significant reduction in the membrane capacitance was observed in cells treated with *SYT13* siRNA and overexpressed with *SYT13* gene in comparison to controls. Data in (a) display the mean ± SD and were analysed by performing a two-sample t-test. Data in (b) are presented as a box-and-whisker plot and were analysed via a Mann–Whitney U test. * *P* < 0.05; ** *P* < 0.01.


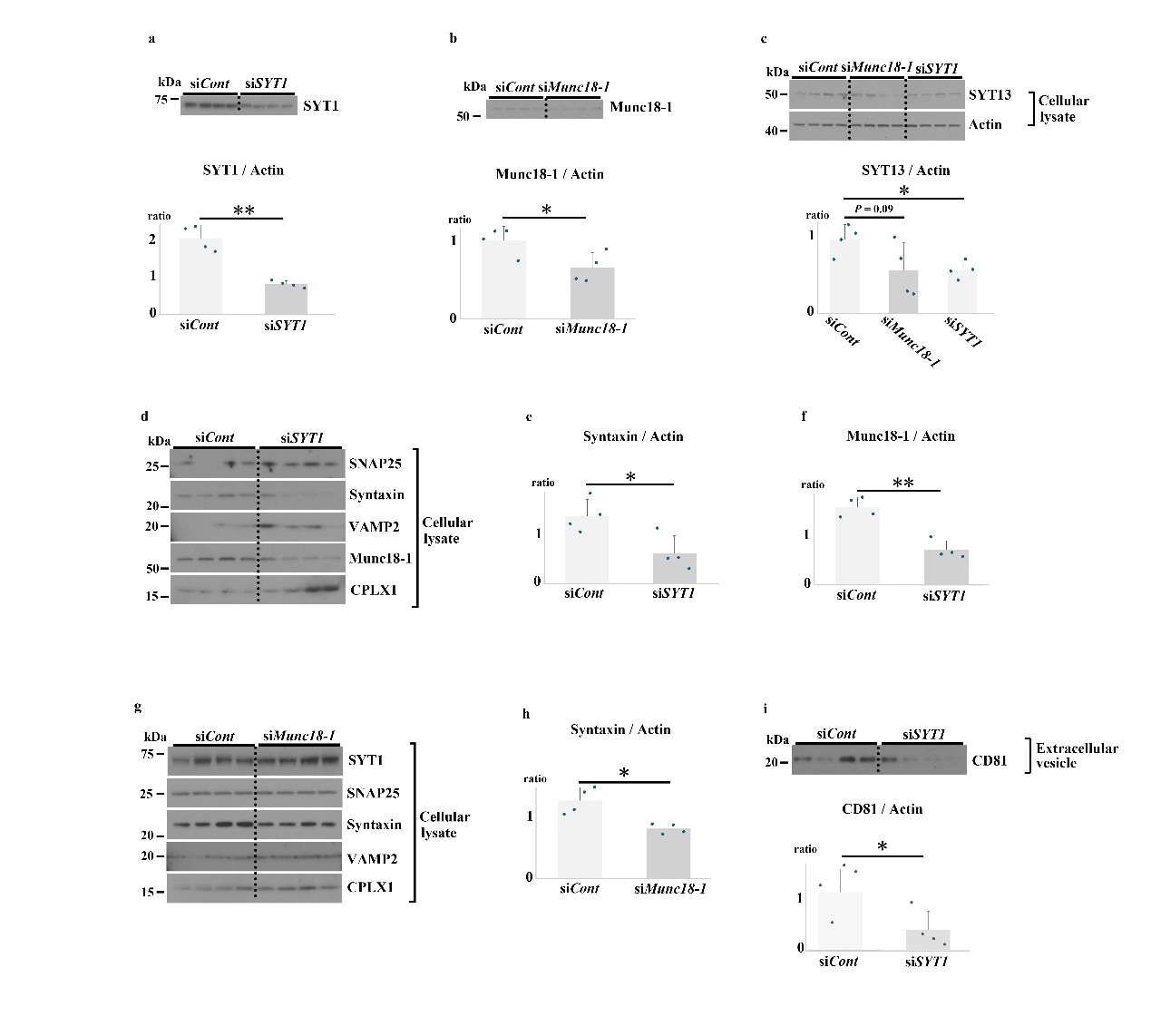


**Fig. S5 Confirmation of interactions between synaptotagmin 13 (SYT13) and SYT1 or mammalian uncoordinated 18-1 (Munc18-1)**

(a–i) The expression of SYT1 or Munc18-1 was reduced in SH-SY5Y cells to investigate the impact on SYT13 and the soluble N-ethylmaleimide-sensitive attachment protein receptor (SNARE) complex protein levels, and the release of extracellular vesicles into the culture supernatant. The knockdown of SYT1 and Munc18-1 in SH-SY5Y cells was achieved through the treatment with siRNA *SYT1* (a) and *Munc18-1* (b), respectively. (c) The administration of *SYT1* siRNA resulted in a notable decline in SYT13 protein levels. The application of *Munc18-1* siRNA also showed a similar trend (*P* = 0.09). (d–h) To further examine the relationship between SYT13, SNARE complexes, and extracellular vesicle release, immunoblotting was performed using SH-SY5Y cells treated with siRNA *SYT1* or siRNA *Munc18-1*. (d–f) The knockdown of SYT1 revealed a significant reduction in the protein levels of Syntaxin and Munc18-1. (g, h) Similarly, the knockdown of Munc18-1 reduced Syntaxin levels. The release of extracellular vesicles was impeded in SH-SY5Y cells treated with *SYT1* siRNA (g, h). For (a–c, e, f, h, i), the protein levels were normalised by actin levels presented in (c). For (a, b, e, f, h, i), a t-test for parametric data was performed, based on the normality of the data evaluated by the Shapiro−Wilk test. For (c), the Shapiro-Wilk test was initially performed, followed by one-way analysis of variance with Tukey’s test. * *P* < 0.05; ** *P* < 0.01


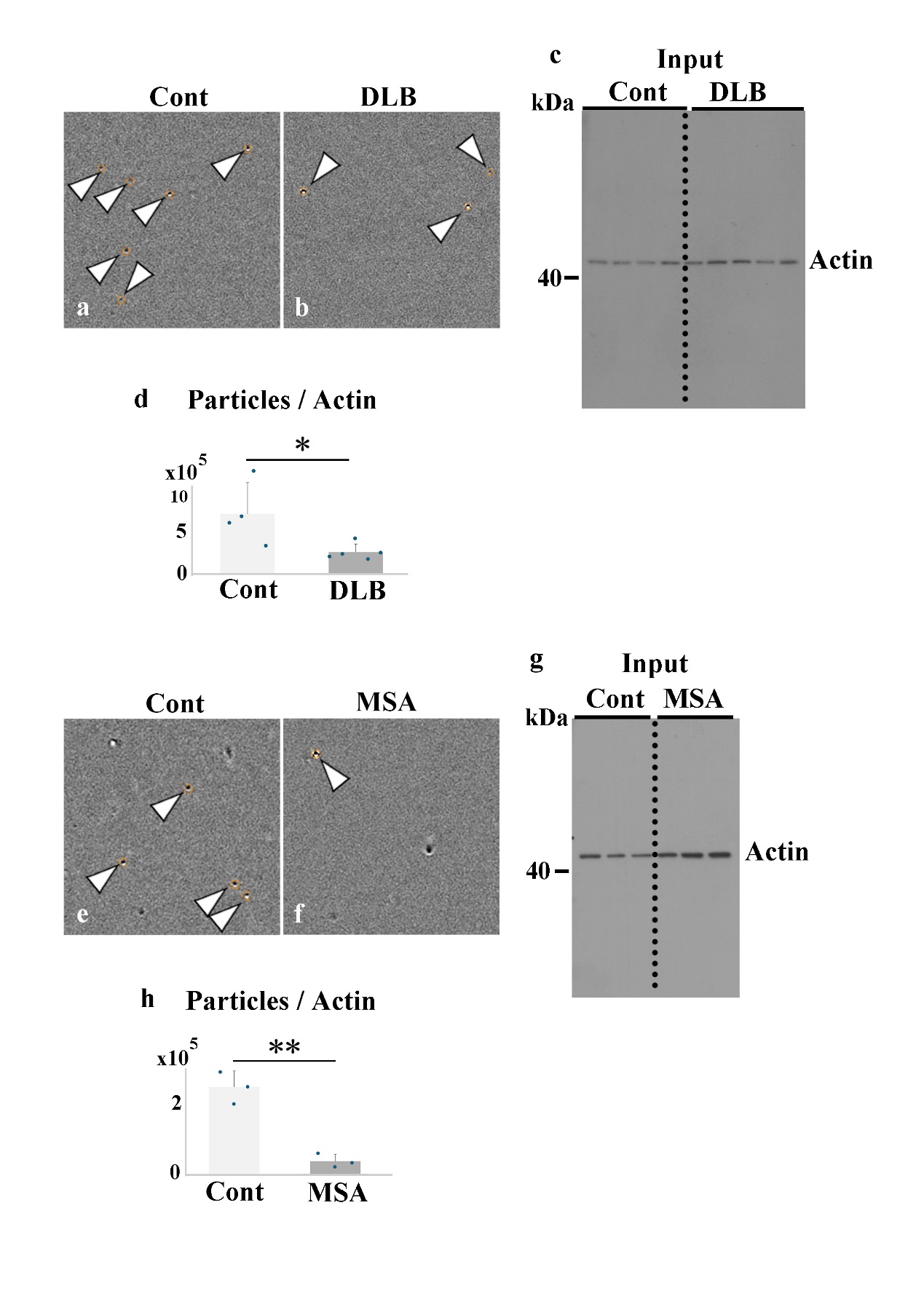


**Fig. S6 Nanoparticle tracking analysis (Video drop) to assess extracellular vesicle release in patients with synucleinopathies.**

(a–g) Nanoparticle tracking analysis (Video drop) was performed to examine the number of extracellular vesicles in the human temporal lobes of patients with dementia with Lewy bodies (DLB: N = 5) and control cases (N = 4), and patients with multiple system atrophy (MSA: N = 3) and control cases (N = 3). Representative images of extracellular vesicles obtained from the brain homogenates of control (a, e), DLB (b), and MSA cases (f). The arrowheads indicate the extracellular vesicles. In each case, the number of extracellular vesicles was normalised relative to the levels of actin (c, g). The ratios of extracellular vesicles to actin were found to be significantly reduced in the samples from the DLB (d) and MSA (h) cases, compared with those in observed in the samples from the control groups. * *P* < 0.05; ** *P* < 0.01


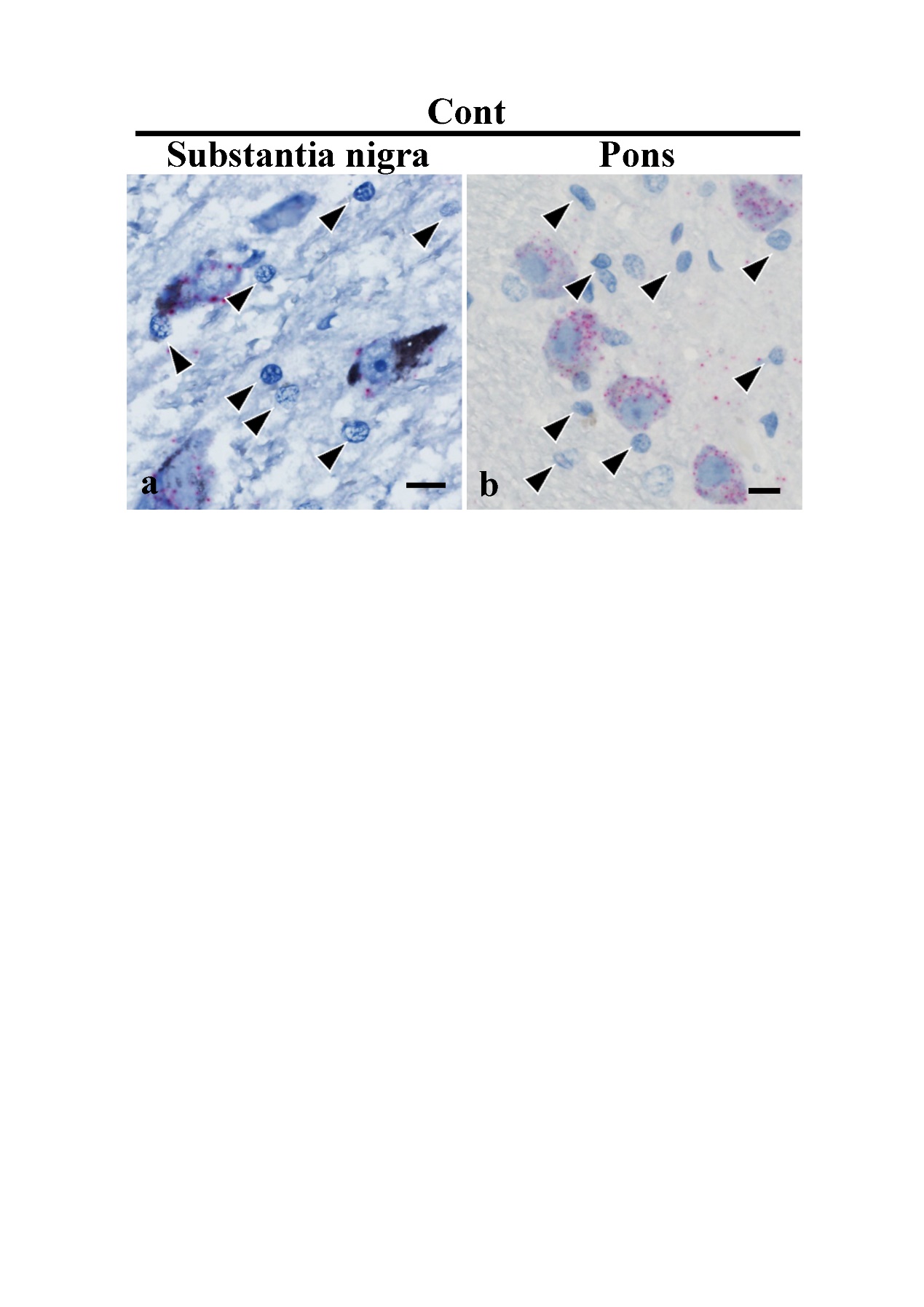


**Fig. S7 mRNA encoding *Synaptotagmin 13 (SYT13)* is expressed in neurons of the human brain.**

RNAscope was performed to identify the localisation of *SYT13* mRNA in the control human brain (N = 4). (a, b) *SYT13* mRNA was observed in neurons, but not in glial cells, including astrocytes, oligodendrocytes, and microglia (arrowheads). Bars = 10 μm (a, b).
