# Supplementary material for: Abnormal α-synuclein binds to synaptotagmin 13, impairing extracellular vesicle release in synucleinopathies
Source: Transl Neurodegener. 2025 Jun 23;14:32. doi: 10.1186/s40035-025-00493-6 (PMC12183919; doi:10.1186/s40035-025-00493-6)
Supplement: Supplementary file 7 — Additional file 7. Predicted interaction between wild type α-Syn and SYT13. Predicted interaction between S129E α-Syn and SYT13 [file 40035_2025_493_MOESM7_ESM.pptx]

## Slide 1
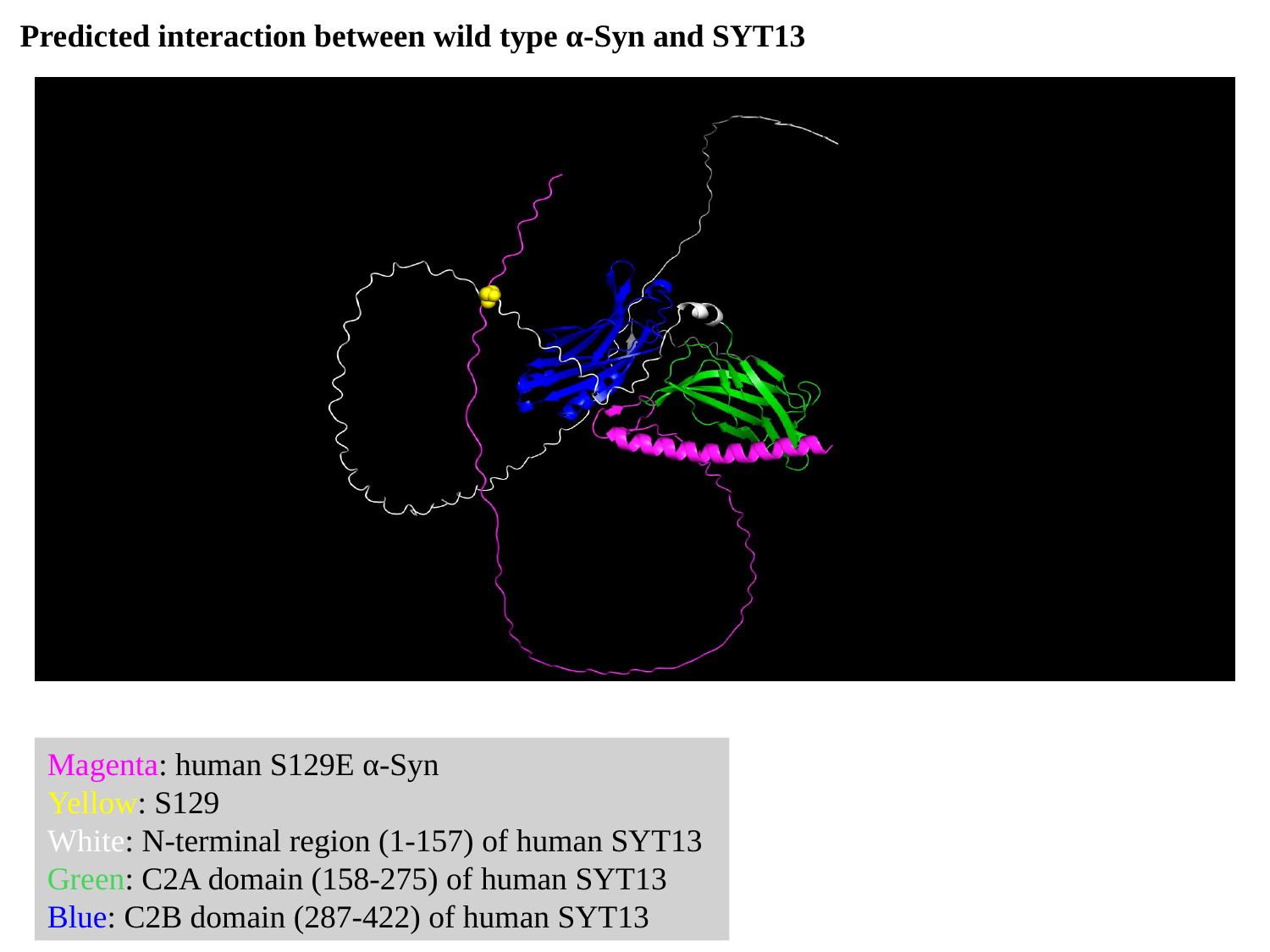

Predicted interaction between wild type α-Syn and SYT13
Magenta: human S129E α-Syn
Yellow: S129
White: N-terminal region (1-157) of human SYT13
Green: C2A domain (158-275) of human SYT13
Blue: C2B domain (287-422) of human SYT13

## Slide 2
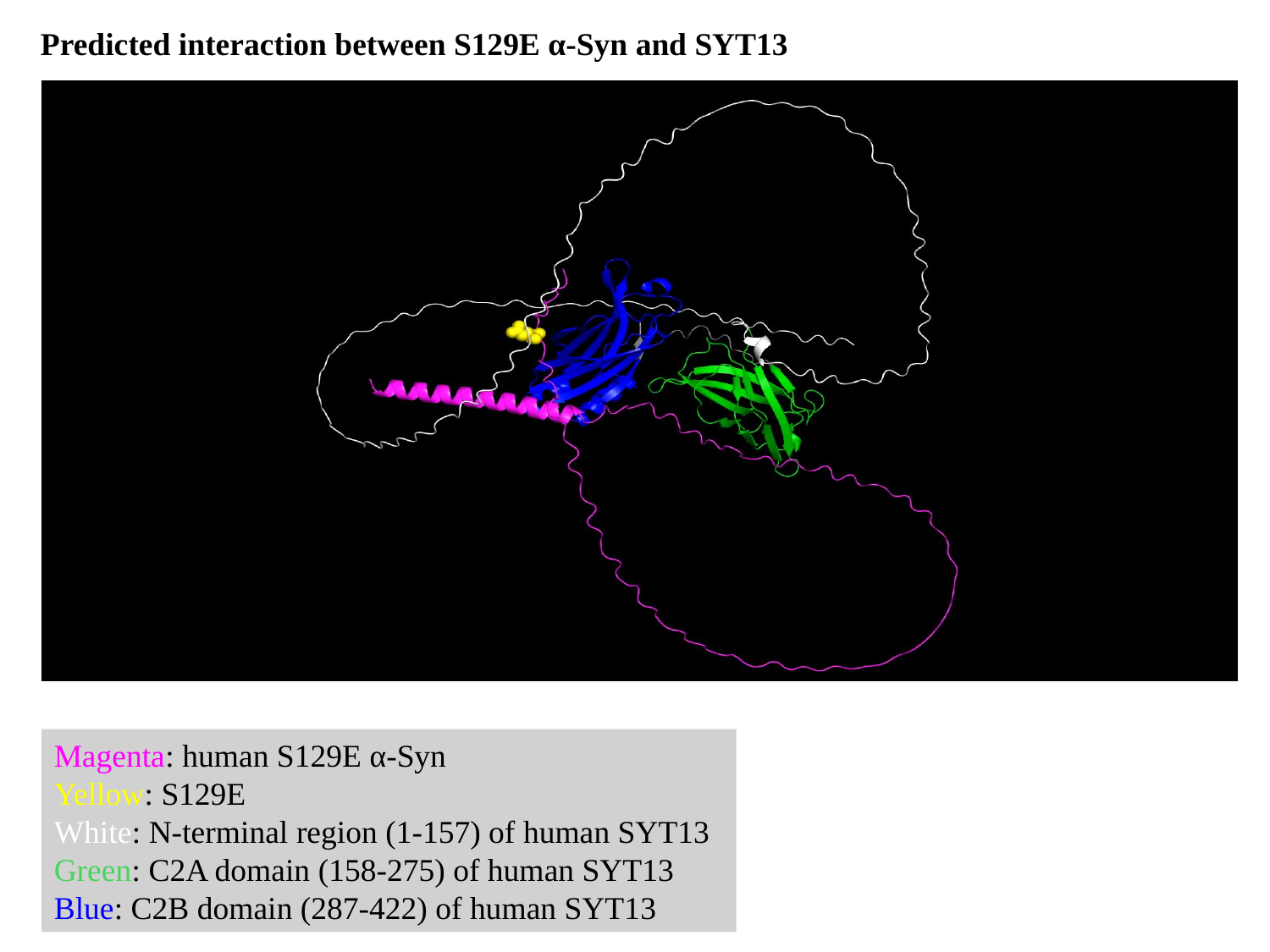

Predicted interaction between S129E α-Syn and SYT13
Magenta: human S129E α-Syn
Yellow: S129E
White: N-terminal region (1-157) of human SYT13
Green: C2A domain (158-275) of human SYT13
Blue: C2B domain (287-422) of human SYT13
