# Supplementary material for: Abnormal α-synuclein binds to synaptotagmin 13, impairing extracellular vesicle release in synucleinopathies
Source: Transl Neurodegener. 2025 Jun 23;14:32. doi: 10.1186/s40035-025-00493-6 (PMC12183919; doi:10.1186/s40035-025-00493-6)
Supplement: Supplementary file 8 — Additional file 8. Uncropped films. [file 40035_2025_493_MOESM8_ESM.pdf]

Fig. 3s uncropped films

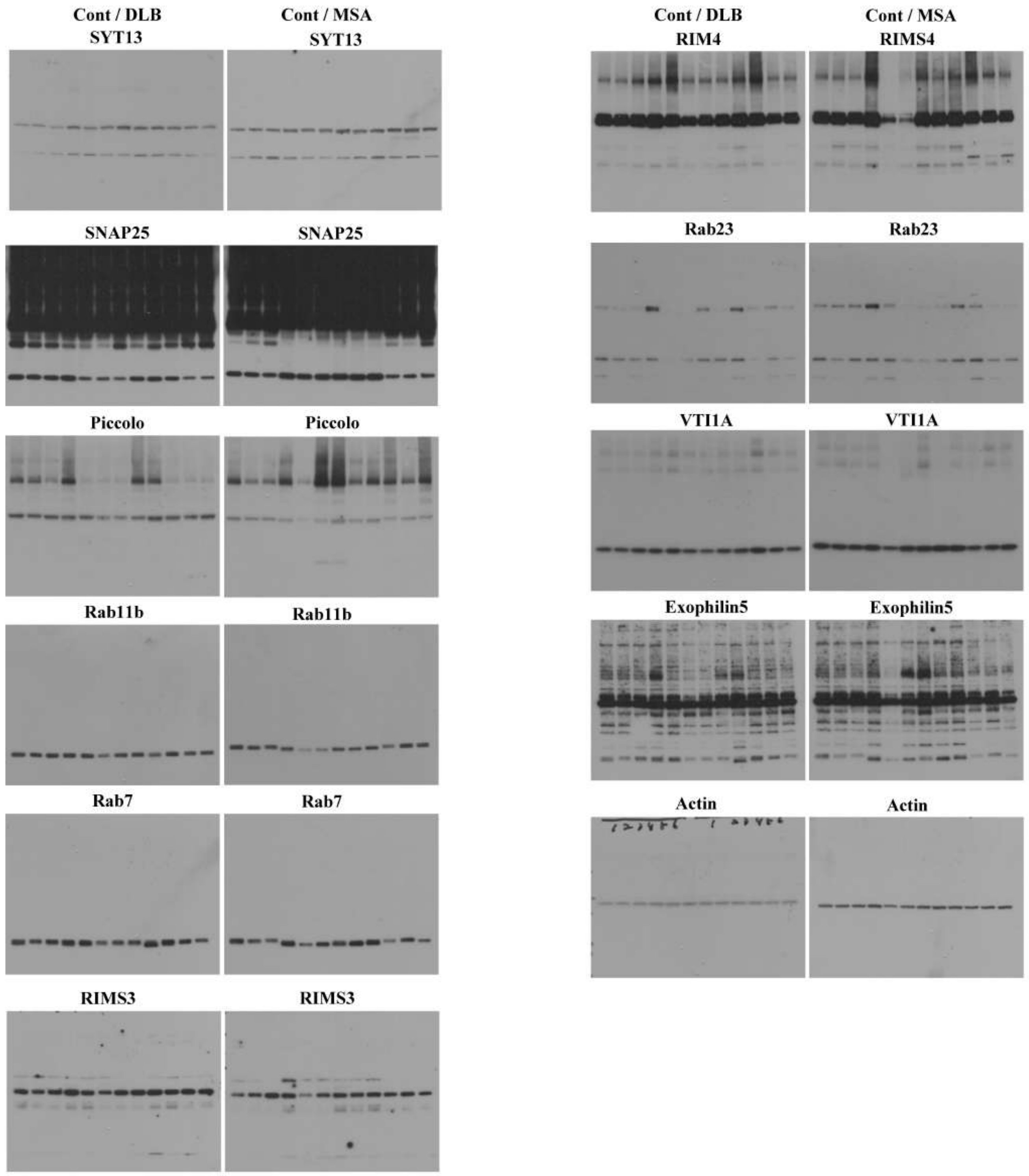

Fig. 5a uncropped films

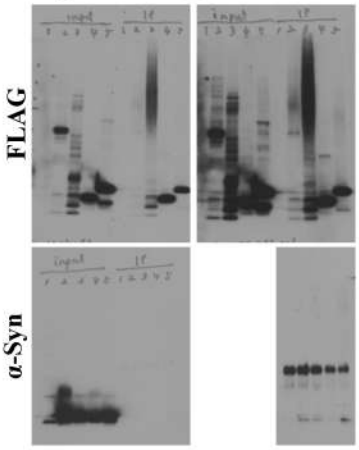

The IP samples were reblotted
for a long exposure version only.

Fig. 6a uncropped films

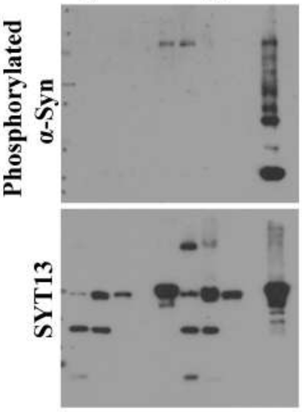

Fig. 5b uncropped films

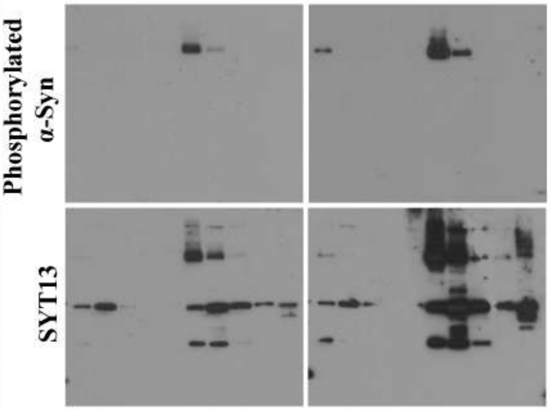

Fig. 6c, d, e: All uncropped films are shown in Fig. 5e, f, g.

Fig. 5b uncropped films

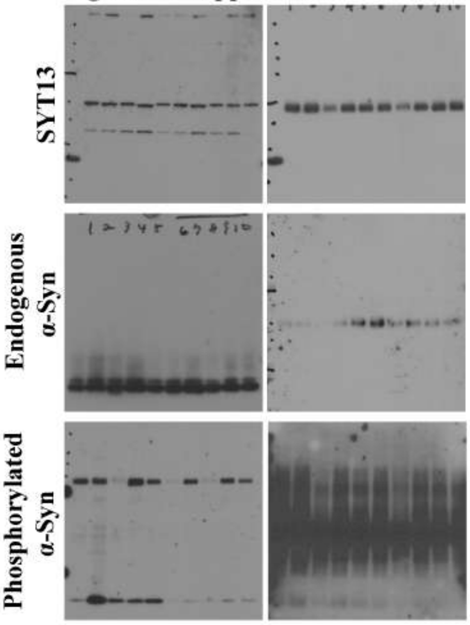

Fig. 5c uncropped films

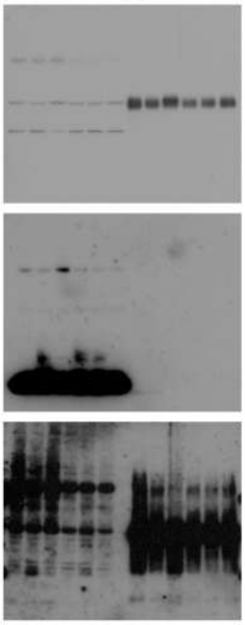

Fig. 7a: The uncropped film is shown in Fig. 7a

Fig. 7b: uncropped films

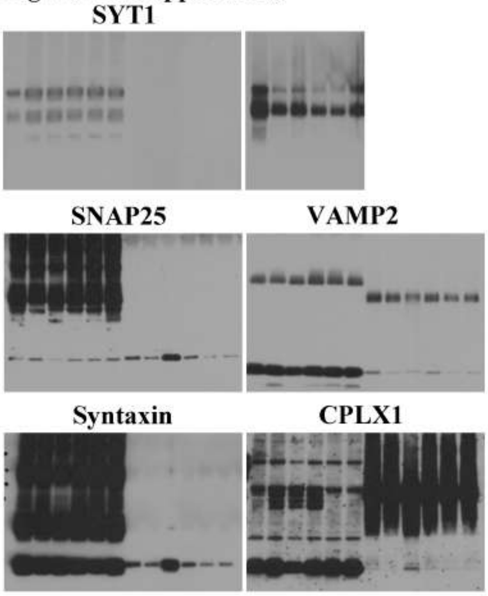

Fig. 7h uncropped films

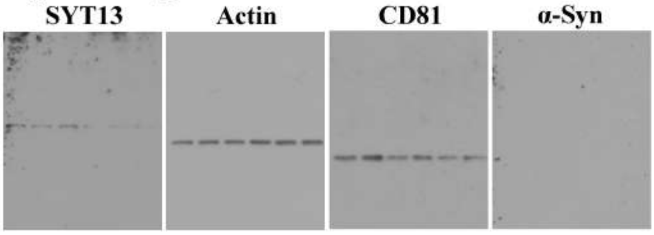

Fig. 7k uncropped films

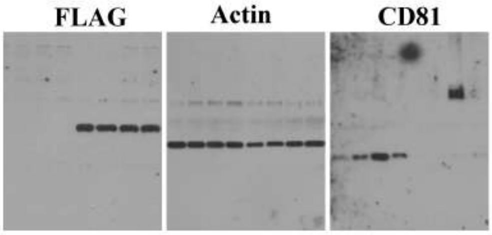

Fig. 7c uncropped films

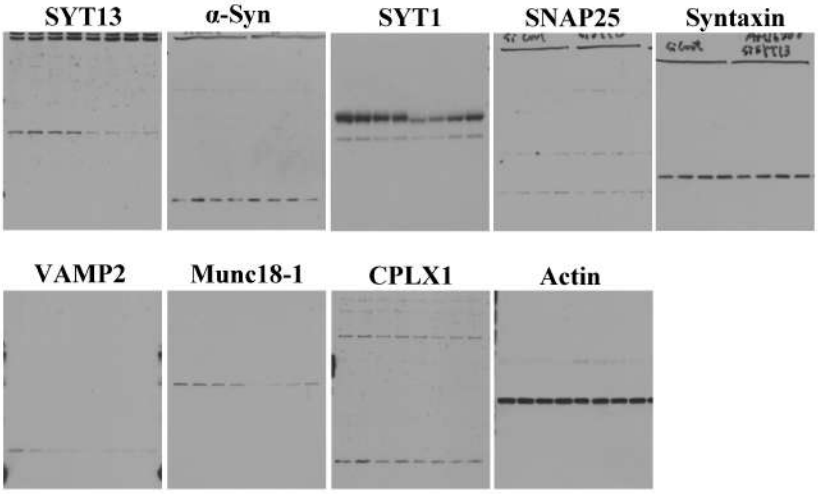

Fig. 8a uncropped films.
An uncropped film regarding 5G4 is shown in Fig. 8a

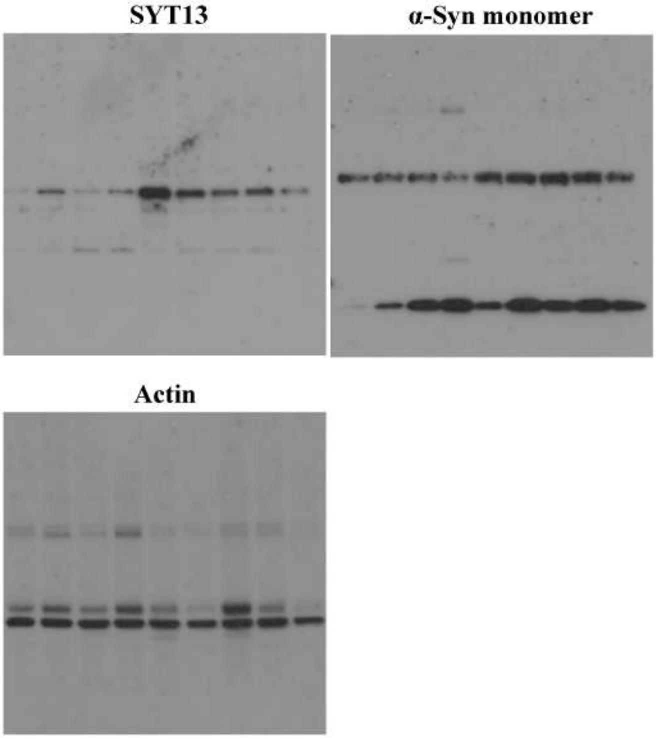

Fig. 8g uncropped films.
An uncropped film regarding 5G4 is shown in Fig. 8g

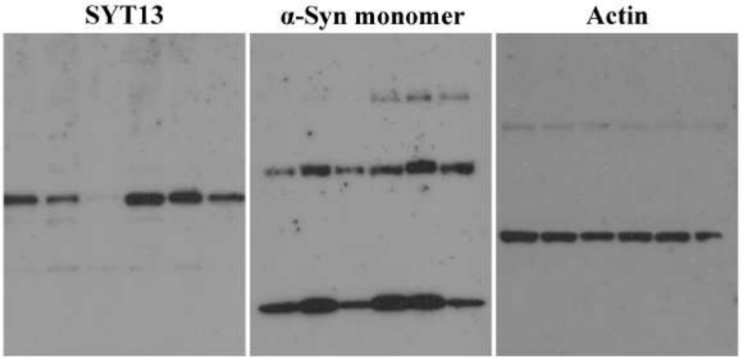

Fig. 7f uncropped films

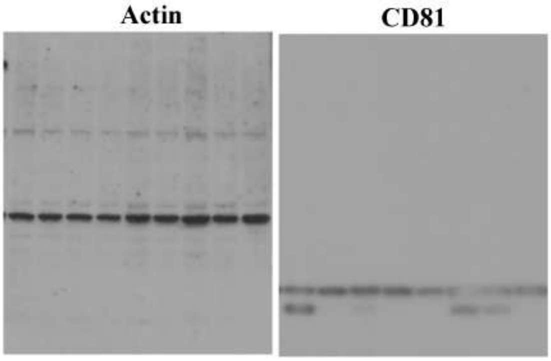

Fig. 7l uncropped films

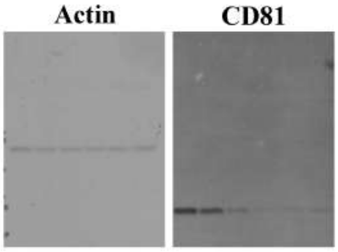

Supplementary Fig. 1a uncropped film

CD81

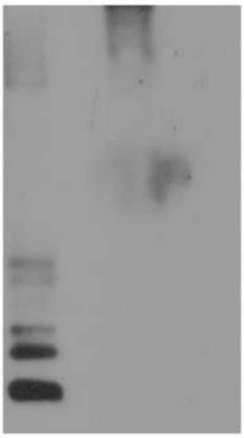

Supplementary Fig. 1b uncropped films

CD81

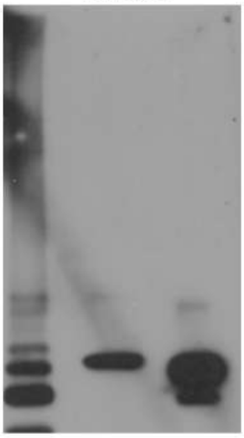

CD9

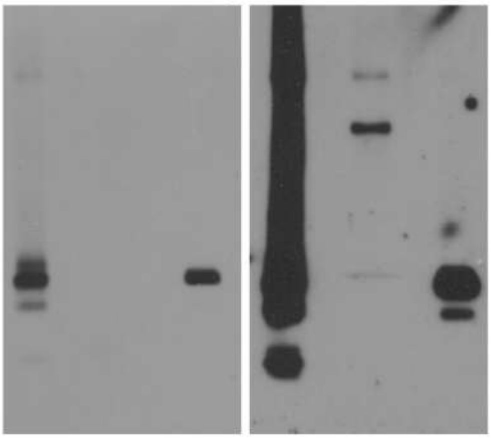

CD63

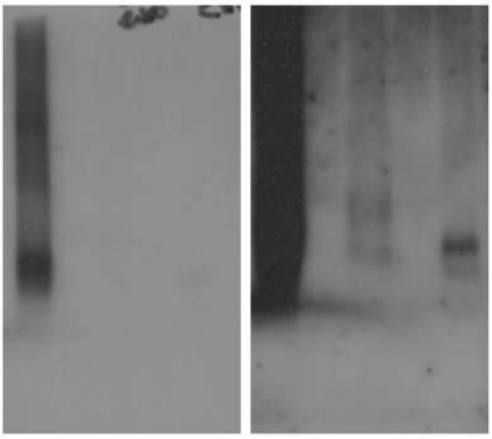

Supplementary Fig. 2

All uncropped films are shown in supplementary Fig. 2

Supplementary Fig. 4a,b uncropped films

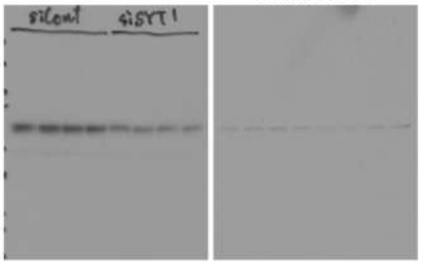

Supplementary Fig. 4c uncropped films

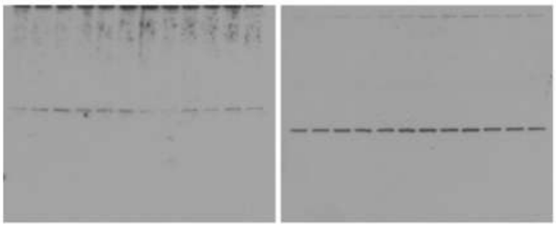

Supplementary Fig. 4d uncropped films

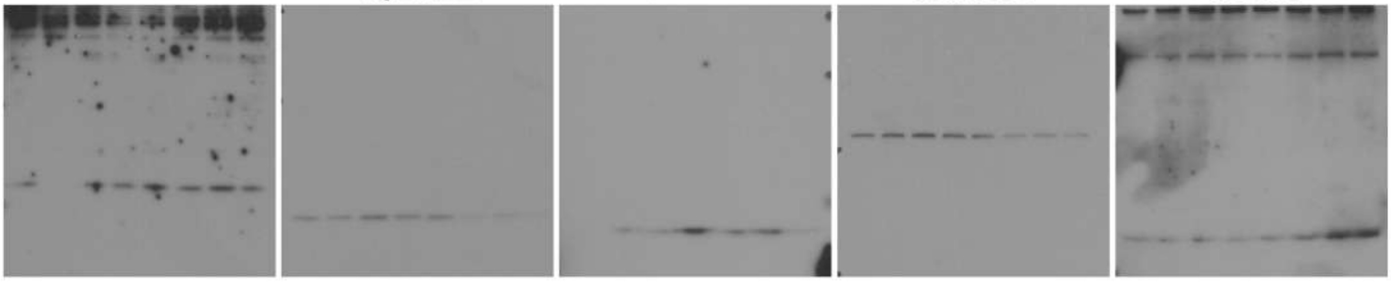

Supplementary Fig. 4g uncropped films

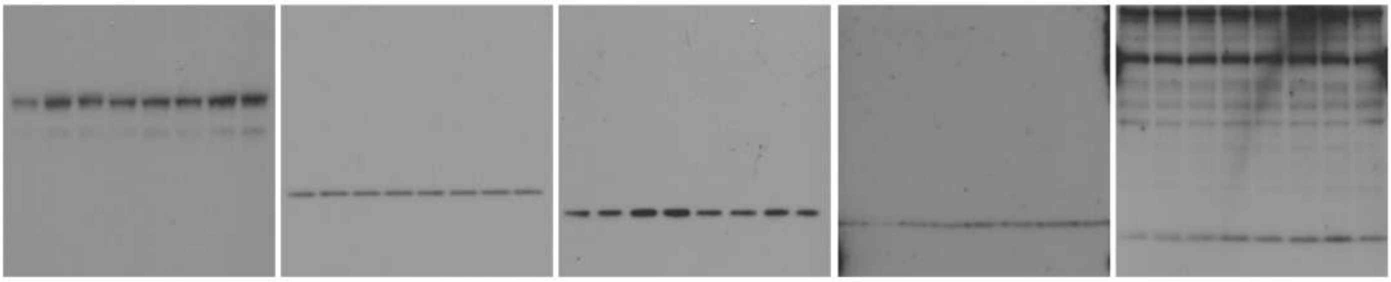

Supplementary Fig. 4i uncropped film

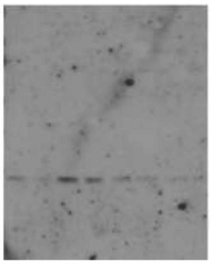

Supplementary Fig. 5: All uncropped films are shown in Supplementary Fig.4 c, g.
